# Microglial TIA1-mediated stress granules promote neuroinflammation and aggravate neuron loss in mice after ischemic stroke by inhibiting IGF2 signaling

**DOI:** 10.7150/thno.122008

**Published:** 2026-01-01

**Authors:** Yiming Qian, Hong Yu, Jianhong Dong, Jinyuan Liu, Jiaqi Han, Tianwen Zheng, Wei Zhang, Lipei Wang, Zhihui Huang, Ying Wang

**Affiliations:** 1 Department of Clinical Research Center, Affiliated Hangzhou First People's Hospital, Westlake University, School of Medicine, Hangzhou 310006, Zhejiang, China.; 2 School of Pharmacy, Hangzhou Normal University, Hangzhou 311121, Zhejiang, China.; 3 School of Basic Medical Sciences, Hangzhou Normal University, Hangzhou 311121, Zhejiang, China.; 4 Department of Pharmacy, Sir Run Run Shaw Hospital, Medical College of Zhejiang University, Hangzhou 310003, Zhejiang, China.; 5 Department of Pharmacy, Affiliated Hangzhou First People's Hospital, School of Medicine, Westlake University, Hangzhou, 310006, Zhejiang, China.

**Keywords:** ischemic stroke, stress granules, neuroinflammation, TIA1, IGF2

## Abstract

**Rationale:** Microglia cells as niche homeostasis monitor with rapid responses to acute ischemic stroke (IS). T-cell intracellular antigen 1 (TIA1), a core component of stress granules (SGs), is involved in cellular stress responses such as hypoxia, but its roles and mechanisms in regulating microglial responses during IS remain unclear.

**Methods:** To evaluate the function of microglial TIA1 in IS, we established a mouse model of IS by using photothrombotic method. Furthermore, conditional knockout (CKO) of *Tia1* in microglia mice (*Tia1*^Cx3cr1^-CKO mice) was generated and then *Tia1*^Cx3cr1^-CKO IS mice and their littermate controls (*Tia1*^f/f^ IS mice) were used as experimental subjects. The behavioral tests, immunostaining, Laser speckle contrast imaging (LSCI), TTC staining, Nissl staining, quantitative real-time PCR (qPCR) and Western blotting were used to assess the effects of microglial *Tia1* deletion in IS progression. *In vitro,* we utilized the microglia cell line (HMC3 cells) and primary cultured microglia to establish an OGD model, and generated stable *TIA1*-knockdown or *TIA1*-overexpressing HMC3 cell lines, and employed a co-culture system of HMC3 and N2a cells to further explore the roles of microglial *Tia1* signaling in IS. Through RNA sequencing (RNA-seq) of control HMC3 cells and *Tia1*-knockdown HMC3 cells, we investigated in depth the role and molecular mechanism of TIA1-mediated insulin-like growth factor 2 (IGF2) signaling pathway in microglia during IS progression.

**Results:** Microglial TIA1 was significantly upregulated in mice during the acute phase of IS. Microglial *Tia1* knockout suppressed microglial pro-inflammatory responses, enhanced anti-inflammatory responses, promoted phagocytic clearance of infarct debris, alleviated neuronal death, and improved motor deficits in post-IS mice.* In vitro*, TIA1 promoted pro-inflammatory responses to exacerbate neuronal cell death and inhibited phagocytic ability of microglia cells after OGD. Mechanistically, *Tia1* deletion in microglia impaired SG formation, reduced sequestration of *Igf2* mRNA into SGs, upregulated IGF2 expression, and IGF2 signaling enhanced anti-inflammatory responses and phagocytic capacity while suppressing pro-inflammatory activation in microglia.

**Conclusions:** These findings identify a previously unrecognized function of microglial TIA1 in modulating microglia homeostasis and sustaining pro-inflammatory responses via SGs-mediated* Igf2* mRNA sequestration after IS, providing a novel therapeutic target for IS treatment.

## Introduction

Stroke, an acute neurological disorder caused by cerebrovascular disruption, remains a leading global cause of adult mortality and long-term disability. Given the limited therapeutic options available, this devastating condition affected approximately 13.7 million people worldwide in 2019, resulting in 5.5 million lives as reported in recent epidemiological studies 1-3. Clinically, stroke manifests in two primary forms based on vascular pathology: hemorrhagic stroke resulting from cerebrovascular rupture with parenchymal hemorrhage, and IS arising from critical hypoperfusion secondary to vascular occlusion. Notably, IS constitutes more than 60% of all cerebrovascular events, and its current interventions including intravenous thrombolysis and endovascular thrombectomy exhibit stringent time-dependency, with treatment efficacy sharply declining beyond narrow therapeutic windows 4.

Cerebral ischemia causes immediate structural damage at the site of the lesion, triggering neuronal dysfunction and progressing to irreversible cell death 5, 6. Concurrently, sustained neuroinflammatory cascades initiated by ischemic neurons significantly influence the progression and clinical outcomes in IS 7, 8. Microglia, the resident immune cells in the central nervous system (CNS), are quickly activated and serve as central mediators in the neuroinflammatory response. These cells undergo polarization into two classical subtypes, the conventionally activated and alternatively activated types, which exert dual effects on neurological damage and recovery 9. Conventionally activated microglia promote secondary brain damage, driving pro-inflammatory responses via cytokine release 10, while alternatively activated microglia promote tissue remodeling through angiogenesis stimulation and anti-inflammatory mediator secretion 9, 11, 12. This phenotypic plasticity suggests that inducing microglial conversion to alternatively activated type, thus developing a promising therapeutic approach to reduce neuroinflammation for enhancing post-stroke recovery.

Following an ischemic insult, cells undergo endoplasmic reticulum (ER) stress, which enhances ER protein-folding and degradative capacities while blocking global synthesis of proteins by promoting the formation of SGs, membraneless ribonucleoprotein condensates containing RNA binding protein (RBPs), translationally arrested mRNAs and initiation factors 13, 14. TIA1, an RBP and core component of SGs, participates in mRNA splicing and is translocated to SGs to regulate gene expression 15-17. Emerging evidence indicates that TIA1 sequesters target mRNAs into SGs during stress, withholding them from ribosomal translation to conserve cellular energy and facilitate damage repair 18-21. Previous studies have shown that SGs formation dynamically happens and evolves in the early stage of IS 22, 23. However, its roles and mechanisms in regulating microglial responses during IS remain unclear.

IGF2 is a pleiotropic factor expressed in CNS, involved in physiological processes such as neurogenesis and the maintenance of immune homeostasis 24. Dysregulation of its expression has been linked to CNS disorders including anxiety and Alzheimer's disease 25, 26. Evidence suggests that IGF2 signaling can influence the activation state of microglia, potentially shifting their polarization toward an anti-inflammatory and pro-repair phenotype 27. This alternative activation is crucial for mitigating secondary neuronal damage and promoting neurological recovery. However, the upstream molecular mechanisms that precisely regulate IGF2 expression and subsequently affect microglial homeostasis remain poorly understood during the acute phase of IS. A deeper understanding of this regulatory axis is essential for developing novel therapeutic strategies for IS that harness the neuroprotective potential of microglia.

In this study, we found that microglial TIA1 was upregulated during the early phase of IS in mice. Conditional ablation of *Tia1* in microglia conferred neuroprotection by ameliorating motor deficits and reducing IS damage by enhancing alternatively activated polarization, phagocytic clearance of cellular debris, and attenuating neuroinflammation in mice after IS. Mechanistically, *Tia1* deletion disrupted SGs assembly, leading to IGF2 upregulation that drove microglial alternative activation. Pharmacological inhibition of IGF2 abolished the neuroprotective benefits in *Tia1*^Cx3cr1^-CKO mice following IS. Collectively, our findings reveal a novel pathway wherein to modulate microglia homeostasis and sustain pro-inflammatory responses via SGs-mediated control of *Igf2* mRNA sequestration, establishing TIA1 as both a critical neuroinflammation regulator and a promising therapeutic target for IS.

## Materials and Methods

### Animals

The floxed *Tia1* allele (*Tia1*^f/f^) mouse line was established through systematic intercrossing of *Tia1*^f/w^ heterozygotes (Shanghai Biomodel Organism Science & Technology Development Co., Ltd.) as described previously 28, 29. *Tia1*^Cx3cr1^-conditional knockout (CKO) mice were generated by crossing *Tia1*^f/f^ mice with *Cx3cr1-Cre* transgenic mice (kindly provided by Prof. Gang Chen, School of Medicine Zhejiang University), which express Cre recombinase in mononuclear macrophage under the control of *Cx3cr1* promoter and conditional knockout genes in mononuclear macrophage including microglia. Genomic DNA was extracted from tail biopsies and genotyped using PCR primer sets (P1: 5'-GAGGCATCAGAATTGTTTTAGTG-3', P2: 5'-GAGATTCTGCGGGGCGATAG-3'), which generated a wild-type (WT) band at 538 bp and a mutant band at 677 bp. Flp was isolated by crossing *Tia1*^f/w^ and Flp mice with wild-type mice. Genomic DNA from mice tail biopsies were genotyped using PCR primer sets (P1: 5'-GTGTTTTCTCCCGCTTGCTG-3'; P2: 5'-AGTGATGCTCTTGGGCTTCC-3'; P3: 5'-ACCAACAGGGTTCCAAGCAA-3'; P4: 5'-GTTGTTCAGCTTGCACCAGG-3'), which generated a WT band at 403 bp and a mutant band at 542 bp. All mouse lines were maintained on a C57BL/6J genetic background.

All mice were housed in a specific pathogen-free (SPF) facility at the Animal Experiment Center of Hangzhou Normal University, maintained on a 12 h light/dark cycle with a standard diet (temperature 22±2 ℃, humidity 50-60%). For all experiments, at least three pairs of 6-8 weeks male mice from the same cage were used. All animal experiments were approved by the Animal Ethical and Welfare Committee of Hangzhou Normal University (Approval No. HSD-20241217-04).

### Cell culture and oxygen-glucose deprivation (OGD) model

The Human Microglia Clone 3 cell line (HMC3 cells, #CRL-3304, RRID: CVCL_II76) and Neuro-2a cell line (N2a cells, #CCL-131, RRID: CVCL_0470) were obtained from the American Type Culture Collection (ATCC, USA). Both cell lines were cultured in DMEM (Gibco, USA) supplemented with 10% FBS (SERANA, Germany) and 1% penicillin-streptomycin, and maintained in an incubator (37 ℃ with 5% CO_2_). The exact purchase date cannot be traced due to incomplete records, but the cell line was authenticated and confirmed to be contamination-free (e.g., mycoplasma negative) prior to experimentation, ensuring the validity of the results.

Primary cultured microglia were isolated from postnatal day 1~3 (P1~P3) C57BL/6 mouse pups. Cortical tissues were dissected in ice-cold 1×PBS, minced, and enzymatically digested with 0.25% trypsin-EDTA for 15 min at 37 ℃. Dissociated cells were filtered through a 70-μm strainer (#BS-70-CS, Biosharp) and seeded in DMEM medium supplemented with 10% FBS, 1% penicillin/streptomycin, and 20 ng/mL GM-CSF (#315-03, PeproTech). Cultures were maintained at 37℃ with 5% CO_2_ for 10~14 days. Microglia were detached by being digested with 0.25% trypsin-EDTA for 25 min 30. Then these primary cultured microglia cells were under OGD treatment or IGF2 treatment (#100-12, PeproTech). To investigate the response of microglia to hypoxia and glucose deprivation *in vitro*, an OGD model was established 31, 32. Briefly, the medium of HMC3 cells or primary cultured microglia was replaced with D-Glucose-free DMEM (#11966025, Gibco, USA) and then the cells were cultured for 4 h in a hypoxia incubator chamber (#27310, Stemcell, Canada) filled with 95% N_2_ and 5% CO_2_ to mimic OGD induced injury.

### Neuron-microglia co-culture

HMC3 and N2a cells were co-cultured using Transwell inserts (#3440, Corning, USA) to investigate the role of microglial TIA1 in regulating neuronal apoptosis following OGD *in vitro*. Briefly, HMC3 cells (4×10^4^ cells) were cultured in the upper chamber, and N2a cells (4×10^5^ cells) were cultured in the lower chamber. The co-culture was subjected to OGD for 4 h, after which apoptosis level of N2a cells was analyzed 32.

### Photothrombotic stroke model (PTS)

Mice were intravenously injected with a rose bengal (#330000, Sigma-Aldrich, USA, 50 mg/kg) solution and allowed to wait for 10 min to ensure the maximum plasma concentration of rose bengal. Afterward, the mouse skull was exposed, and a laser generator (#XD8038, Seepdtech) was positioned above the left motor sensory cortex of the mouse brain (0.5 mm posterior to the bregma and 1.5 mm left to the midline). The laser was used to irradiate this site for 10 min to induce photothrombosis. After the procedure, mice were sutured and returned to their home cages following recovery on a heating pad. The schematic model was shown in Supplementary Figure S1A, and TTC staining (Supplementary Figure S1B) confirmed the successful establishment of the IS model.

### Behavioral analysis

As previously described 33, the following behavioral assays were used to assess the neurobehavioral function of mice after IS injury. All the behavioral experiments were performed on the 1^st^, 3^rd^, 5^th^ and 7^th^ days after IS, with the open field test specifically conducted on the 3^rd^ day after IS, as shown in Supplementary Figure S1A.

### Open field test

All mice were individually placed in a square box (40×40×40 cm^3^) and allowed to explore freely for 15 min in a quiet environment. The total distance traveled by each mouse was recorded as a measure of locomotor activity.

### Grid walk test

A wire grid (40 cm×30 cm) was fixed at a height of 50 cm above the ground, and mice were placed on the grid. A camera was positioned beneath it to record videos. The number of the right forelimb slipped during the first hundred steps (both forelimbs) were recorded.

### Rotarod test

Mice were trained daily on a rotarod apparatus (#SA02M, SANS, Jiangsu) for 5 min over 3 days before IS modeling. During training, the rotarod speed was gradually increased from 0 rpm to 40 rpm. The mice were subsequently tested under the same conditions after IS modeling, and the retention time on the rotarod was recorded and averaged after triplicate experiments.

### Cylinder test

Mice were placed in a transparent cylinder (15 cm in diameter and 30 cm in height) with a video camera positioned below for video recording. The contact of the forelimbs with the wall of the cylinder was recorded during the first 10 min. Mice forelimb asymmetry index was calculated as follows:

Forelimb use asymmetry = (n_left contact_ - n_right contact_) / (n_left contact_ + n_right contact_ + n_both paws contact_)×100%

### Modified neurological severity score (mNSS)

mNSS analysis was used to evaluate the neurological deficits of mice after IS injury, comprising motor, sensory, reflex and balance evaluations with a scoring range of 0 to 18 34. All mice were evaluated by researchers blinded to the experimental conditions. Higher scores indicated more severe neurological deficits.

### Western blotting

The tissues of *Tia1*^f/f^ and *Tia1*^Cx3cr1^-CKO mice were lysed in ice-cold RIPA buffer (#P0013B, Beyotime, Shanghai) containing protease inhibitor at 4℃ for 30 min, centrifuging at 1.4×10^4^ g for 30 min at 4℃ to isolate the total protein. Protein concentrations were determined using a BCA protein analysis reagent (Thermo Fisher Scientific). After being separated by sodium dodecyl sulphate polyacrylamide gel electrophoresis (SDS-PAGE), proteins were transferred to polyvinylidene fluoride transfer membranes (Merck Millipore). Membranes were blocked with 5% skim milk and incubated overnight at 4 ℃ with primary antibodies: anti-Bax (1:5, 000, ET1603-34, Huabio), anti-Bcl-2 (1:2, 000, ET1702-53, Huabio), anti-TIA1 (1:1, 000, #12133-2-AP, Proteintech), anti-CD206 (1:200, #60143-1-Ig, Proteintech), anti-CD206 [1:1, 000, #24595, Cell Signaling Technology (CST)], anti-CD86 (1:1, 000, #bsm-52375R, Bioss), anti-CD86 (1:1, 000, #13395-1-AP, Proteintech), anti-IGF2 (1:1, 000, #ab9574, Abcam), anti-beta Tubulin (1:10, 000, #ET1602-4, Huabio) and anti-GAPDH (1:5, 000, #ET1601-4, Huabio). After washing, membranes were incubated with horseradish peroxidase (HRP)-conjugated secondary antibodies solution at room temperature for 1 h. Finally, chemical signals of the proteins were detected using a Super-sensitive ECL Chemiluminescent Substrate (BL520A, Biosharp) on an imaging workstation (Guangzhou Boluteng Biotechnology Company). The integrated density of the bands was measured by ImageJ software.

### Nissl staining

Mice were euthanized at specified time points following IS. Briefly, mice were transcardially perfused with 1×PBS, followed by 4% PFA. Brain tissues were dissected and soaked in 4% PFA for at least 24 h, then submerged in 30% sucrose solution until the tissues sank. Coronal brain sections (20-μm thickness) were prepared using a cryostat (Thermo, USA). After drying at 60 ℃ for 30 min, sections were incubated with 0.1% cresyl violet for 5 min, rinsed with double-distilled water, and dehydrated through a graded ethanol series (75%, 95%, 100%). Tissues were cleared in xylene and sealed with the neutral resin. Images were acquired with a microscope (Olympus VS200, Japan).

### 2, 3, 5-triphenyltetrazolium chloride (TTC) staining

2% TTC (#T8877, Sigma-Aldrich, US) solution was prepared with normal saline.* Tia1*^f/f^ and *Tia1*^Cx3cr1^-CKO mice were anesthetized and sacrificed on the 3^rd^ day after IS. The brain tissues were immediately removed and snap-frozen at -80℃ within 2 min, and then equally cut into 5 or 6 coronal sections, and the sections were completely immersed in TTC working solution and reacted in the dark at 37℃ for 30 min. TTC working solution was then discarded and the brain sections were immersed in 4% PFA for 30 min to fix. Finally, the brain sections were photographed and ImageJ software was used to analyze them.

### Immunofluorescence staining

Tissue sections were dried at 60 ℃ for 30 min, fixed for 15 min, and immersed in sodium citrate buffer for antigen retrieval (90 ℃, 30 min). After natural cooling, sections were permeabilized and blocked with 5% BSA containing 0.3% Triton X-100 for 1 h. Sections were then incubated overnight at 4 ℃ with the primary antibody solutions, including: guinea pig-anti-Iba1 (1:500, #OB-PGP049, Oasis Biofarm), rabbit-anti-Iba1 (1:500, #ab178846, Abcam), rabbit-anti-TIA1 (1:500, #ab140595, Abcam), mouse-anti-GFAP (1:500, #MAB360, Merck), mouse-anti-NeuN (1:500, #E4M5P, CST), rabbit-anti-iNOS (1:500, #ab178945, Abcam), rabbit-anti-ARG-1 (1:500, #HA721147, Huabio), rabbit-anti-ARG-1 (1:200, #A25808, ABclonal), rabbit anti-cleaved-caspase 3 (1:400, #9661, CST), mouse-anti-G3BP1 (1:500, #66486-1-Ig, Proteintech), mouse-anti-MBP (1:500, #sc-271524, Santa), rabbit-anti-LAMP1 (1:100, #ab208943, Abcam), rabbit-anti-Galectin3 (1:500, #ab76245, Abcam), rabbit-anti-IGF2 (1:500, #ab9574, Abcam). Following washing, sections were incubated with species-matched secondary antibodies for 1 h at room temperature, including: donkey anti-rabbit Alexa Fluor546 (1:1, 000, #A10040, Invitrogen), goat anti-mouse Alexa Fluor546 (1:1, 000, #A11030, Invitrogen), goat anti-rabbit Alexa Fluor488 (1:1, 000, #A32731, Invitrogen), donkey anti-mouse Alexa Fluor488 (1:1, 000, #A21202, Invitrogen), goat anti-guinea pig IgG (H+L) 488 (1:1, 000, #G-GP488, Oasis Biofarm), goat anti-guinea pig IgG (H+L) 647 (1:1, 000, #G-GP647, Oasis Biofarm), goat anti-rabbit Alexa Fluor647 (1:1, 000, #A0468, Beyotime). Finally, a fluorescence microscope (Olympus VS200, Japan) or confocal laser microscopes (Nikon AX or Olympus FV3000) was used to acquire the images, and ImageJ software was used to analyze them.

### TUNEL staining

Double immunofluorescence staining for NeuN and TdT-mediated dUTP Nick-End Labeling (TUNEL) was performed to detect neuronal apoptosis. Firstly, tissue sections underwent NeuN immunofluorescence staining and secondary antibody incubation as described in the immunofluorescence staining protocol above. TUNEL staining was then conducted using a TUNEL Apoptosis Detection Kit (FITC) (#40306ES50, Yeasen, Shanghai) following the manufacturer's protocol. Briefly, sections were incubated in equilibration buffer for 30 min at room temperature, followed by incubation with TdT reaction buffer (prepared according to the kit formula) at 37 ℃ in the dark for 1 h, then mounted after washing. A fluorescence microscope (VS200, Olympus, Japan) was used to acquire the images and ImageJ software was used to analyze them.

### Laser speckle contrast imaging (LSCI)

LSCI was used to assess cerebral blood flow (CBF) dynamics following IS 35. Mice were deeply anesthetized, and their heads were secured in a stereotaxic frame (RWD Life Science Co., Ltd, Shenzhen, China). Erythromycin ophthalmic ointment was applied to protect the eyes from dryness. A midline scalp incision was made to expose the skull, which was then coated with mineral oil to maintain hydration. LSCI measurements were conducted at two time points: baseline (24 h prior to IS induction) and 3 days post-IS. At each time point, continuous CBF monitoring was performed for 5 min. A circular region of interest (ROI, 5 mm diameter) centered on the intended injury site was selected. CBF was measured within the ROI and quantified as perfusion units (PUs).

### Real-Time PCR (RT-PCR)

Total RNA was isolated from the infarct tissues of *Tia1*^f/f^ and* Tia1*^Cx3cr1^-CKO mice on the 3^rd^ day after IS using the RNA-easy isolation reagent (#R701-01-AA, Vazyme, Nanjing). Subsequently, reverse transcription was carried out with the HiScript II One Step RT-PCR Kit (#P611-01, Vazyme, Nanjing). The expression levels of mRNA were quantified by quantitative real-time PCR (qPCR) using the SYBR Green PCR master mix (#Q511-02/03, Vazyme, Nanjing) on a Bio-rad CFX Manager real-time PCR system. Each sample was amplified independently in triplicate. The relative gene expression was calculated using the 2^-ΔΔCt^ method. The primers, synthesized by Qingke Biotech, were listed as followes:

*GAPDH* (human): Forward, 5'-ACAACTTTGGTATCGTGGAAGG-3', Reverse, 5'-GCCATCACGCCACAGTTTC-3'. *GapdH* (mouse): Forward, 5'-AAGAGGGATGCTGCCCTTAC-3', Reverse, 5'-TACGGCCAAATCCGTTCACA-3'. *ACTB* (human): Forward, 5'-CCACCATGTACCCTGGCATT-3', Reverse, 5'-GTCCTCGGCCACATTGTGAA-3'. *Actb* (mouse): Forward, 5'-AGAGCTACGAGCTGCCTGAC-3', Reverse, 5'-AGCACTGTGTTGGCGTACAG-3'. *Ifn-γ* (mouse): Forward, 5'-AGCGGCTGACTGAACTCAGATTGTAG-3', Reverse, 5'-GTCACAGTTTTCAGCTGTATAGGG-3'. *IL-1β* (human): Forward, 5'-AACCTCTTCGAGGCAAGG-3', Reverse, 5'-AGATTCGTAGCTGGATGCCG-3'. *Il-1β* (mouse): Forward, 5'-TGGACCTTCCAGGATGAGGACA-3', Reverse, 5'-GTTCATCTCGGAGCCTGTAGTG-3'. *IL-6* (human): Forward, 5'-ACTCACCTCTTCAGAACGAATTG-3', Reverse, 5'-CCATCTTTGGAAGGTTCAGGTTG-3'. *Il-6* (mouse): Forward, 5'-TAGTCCTTCCTACCCCAATTTCC-3', Reverse, 5'-TTGGTCCTTAGCCACTCCTTC-3'. *IL-4* (human): Forward, 5'-CCAACTGCTTCCCCCTCTG-3', Reverse, 5'-TCTGTTACGGTCAACTCGGTG-3'. *Il-4* (mouse): Forward, 5'-GGTCTCAACCCCCAGCTAGT-3', Reverse, 5'-GCCGATGATCTCTCTCAAGTGAT-3'. *TGF-β* (human): Forward, 5'-GGCCAGATCCTGTCCAAGC-3', Reverse, 5'-GTGGGTTTCCACCATTAGCAC-3'. *Tgf-β* (mouse): Forward, 5'-CTCCCGTGGCTTCTAGTGC-3', Reverse, 5'-GCCTTAGTTTGGACAGGATCTG-3'. *IGF2* (human): Forward, 5'-ACACCCTCCAGTTCGTCTGT-3', Reverse, 5'-GGGGTATCTTGGGGAAGTTGT-3'. *Igf2* (mouse): Forward, 5'-GTTGGTGCTTCTCATCTCTTTG-3', Reverse, 5'-AAACTGAAGCGTGTCAACAAG-3'.

### Plasmid construction and cell transfection

To establish stable *TIA1*-overexpressing HMC3 cells, the *TIA1* cDNA was amplified and inserted into the pLVX-EGFP-IRES-NEO vector using the restriction enzyme sites EcoRI and AgeI. The specific primer sequences used for amplification of *TIA1* cDNA were as follows:

Forward: 5'-CCGGAATTCGCCACCATGGAGGACGAGATGCCCAAG-3', Reverse: 5'-GGCACCGGTCCCTGGGTTTCATACCCTGCC-3'.

The plasmid construction was verified by sequencing.

To establish stable *TIA1*-knockdown HMC3 cells, the *TIA1* shRNA sequence (5'-GCTCTAATTCTGCAACTCTTT-3') was inserted into the pLKO.1 vector using restriction enzyme sites EcoRI and AgeI. The 293T cells were seeded in 6 cm culture dishes, and once reaching 50%~60% confluence, the cells were transfected with mock plasmid, *TIA1*-overexpressing or *TIA1*-knockdown plasmid using Lipofectamine 3000 transfection reagent (#L3000-015, Invitrogen, USA) for 24~72 h. The viral supernatant was then collected and filtered through a 0.22 μm Millipore filter. HMC3 cells were subsequently infected with the filtered viral particles, and transfected cells were harvested for subsequent experiments.

### Phagocytosis analysis *in vitro*

Red fluorescent beads (#L2778, Sigma-Aldrich, US) were used to assess the phagocytic capacity of microglia following OGD *in vitro*. Briefly, after OGD treatment, HMC3 cells were incubated with red fluorescent beads (0.5 μL/mL) for 2 h. Non-phagocytosed beads were then removed by washing with 1×PBS, and a fluorescence microscope (#CKX53, Olympus, Japan) was used to acquire the images.

### RNA sequencing analysis

After OGD for 4 h, total RNA was extracted from HMC3 cells using RNAiso Plus reagent (Takara, Japan) and subjected to whole RNA sequence analysis by Novogene Corporation (Beijing, China). RNA quantity and integrity were evaluated using the RNA Nano 6000 Assay Kit on a Bioanalyzer 2100 system (Agilent Technologies, CA, USA). Resulting *P*-values were adjusted via the Benjamini-Hochberg approach to control for false discovery rate. *P*-value < 0.05 and |log_2_(FoldChange)|>1 were set as the threshold for significantly differential expression. Data were uploaded to the GSE297158 dataset in the Gene Expression Omnibus database.

### RNA immunoprecipitation (RIP)

Briefly, the steps were as follows: peri-infarct cortical tissues of *Tia1*^f/f^ mice at 3 days after IS were collected. These tissues were processed in accordance with the manufacturer's instructions (Cat. Bes5101, BersinBio) and our previous protocol 29. Subsequently, the expression level of *Igf2* mRNA were measured via qRT-PCR. The primer sequences for *Igf2* were provided in the Real-Time PCR section.

### Statistical analysis

All the data were presented as mean ± s.e.m. and analyzed by GraphPad Prism 8. Student's *t*-test was used to determine the statistical significance between two groups, one or two-way ANOVA was used to determine the statistical significance between multiple groups. Mann-Whitney test was performed to analyze the data that were not normally distributed. The results with a *P*-value < 0.05 were considered statistically significant. All experiments were performed at least three independent times.

## Results

### TIA1 was significantly upregulated in microglia during acute phase of IS, and TIA1 deficiency in microglia alleviated the motor behavioral deficits in mice after IS

**Figure 1:**
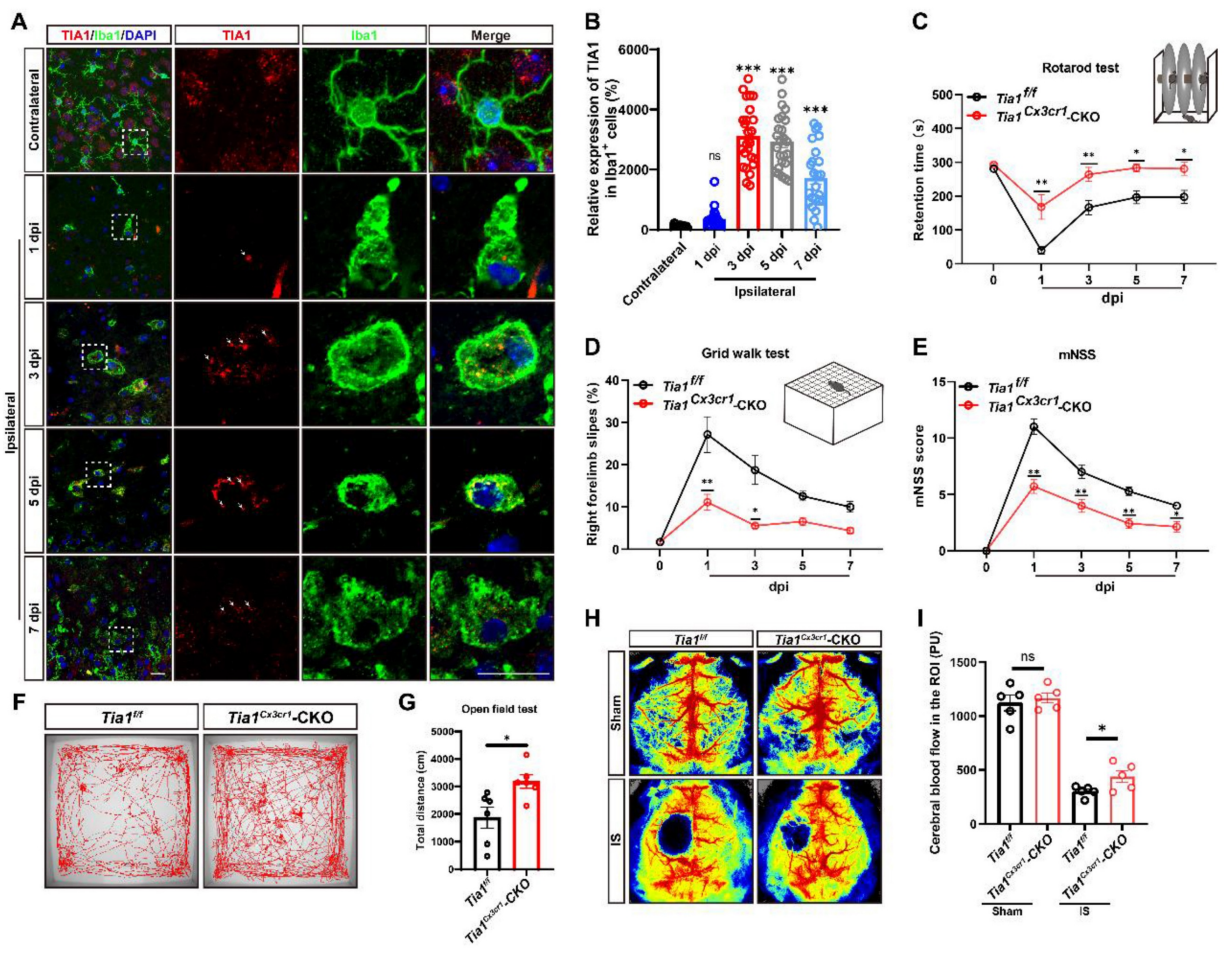
** TIA1 was upregulated in microglia after IS, and *Tia1* deficiency in microglia alleviated the motor behavioral deficits in mice after IS.** (**A**) Immunostaining analysis of TIA1 (red) and Iba1 (green) in contralateral (1^st^ day) and infarct area of *Tia1*^f/f^ mice on the 1^st^, 3^rd^, 5^th^ and 7^th^ day after IS. Scale bars, 20 µm. (**B**) Quantitative analysis of the relative expression of TIA1 in Iba1^+^ cells as shown in (**A**) (n = 25 cells). (**C-D**) Behavioral analysis of *Tia1*^f/f^ and* Tia1*^Cx3cr1^-CKO mice by rotarod test (**C**, n = 7 mice per group) and grid walk test (**D**, n = 7 mice per group) after IS. (**E**) Quantitative analysis of mNSS scores of *Tia1*^f/f^ and* Tia1*^Cx3cr1^-CKO mice after IS (n = 7 mice per group). (**F**) Representative tracing images of the open field test of *Tia1*^f/f^ and* Tia1*^Cx3cr1^-CKO mice on the 3^rd^ day after IS. (**G**) Quantitative analysis of the total moved distance of *Tia1*^f/f^ and* Tia1*^Cx3cr1^-CKO mice in the open field test on the 3^rd^ day after IS as shown in (**F**) (n = 6 mice per group). (**H**) Representative images taken by LSCI of regional CBF in the cortical region of *Tia1*^f/f^ and* Tia1*^Cx3cr1^-CKO mice on the 3^rd^ day after IS. (**I**) Quantitative analysis of CBF changes before and after IS as shown in (**H**) (n = 5 mice per group). Data were shown as mean ± s.e.m. *ns = no significance*, ^*^P < 0.05, ^**^P < 0.01, ^***^P < 0.001, compared with control.

To investigate the potential role of microglial TIA1 in IS, the temporal and spatial expression pattern of TIA1 in microglia was first examined using a PTS mouse model (Supplementary Figure S1). Interestingly, TIA1 was significantly upregulated in Iba1^+^ microglia on the 3^rd^ day after IS, with sustained expression levels through the 7^th^ day (Figure 1A-B), suggesting that IS induced the upregulation of TIA1 in microglia during the acute phase of IS. To further explore the role of microglial TIA1 in IS, *Tia1*^Cx3cr1^-CKO mice (conditional knockout of *Tia1* in microglia) were generated by crossing *Tia1*^f/f^ with *Cx3cr1-Cre* transgenic mice (Supplementary Figure S2A-B). Indeed, TIA1 was significantly reduced in microglia of *Tia1*^Cx3cr1^-CKO mice on the 3^rd^ day after IS (the time point of peak TIA1 expression in wild-type microglia) (Supplementary Figure S2C). No significant differences were observed in body size or weight between 2-month-old *Tia1*^f/f^ and *Tia1*^Cx3cr1^-CKO mice (Supplementary Figure S2D-E). Concurrently, a series of behavioral tests, including the open field test, cylinder test and rotarod test showed no significant differences in motor functions such as bilateral limb coordination and basic locomotor ability between 2-month *Tia1*^f/f^ and *Tia1*^Cx3cr1^-CKO mice (Supplementary Figure S2F-I), suggesting that *Tia1* knockout in microglia did not substantially affect mouse development or motor function.

However, after IS, the motor behavioral deficits were significantly alleviated in* Tia1*^Cx3cr1^-CKO mice. For instance, *Tia1*^Cx3cr1^-CKO mice stayed on the rotarod for a longer time (Figure 1C), had fewer forelimb deficits in the grid walk test (Figure 1D), and covered longer distances in the open field test after IS (Figure 1F, G). Additionally, mNSS results also indicated that *Tia1*^Cx3cr1^-CKO mice had a better motor status after IS (Figure 1E). Finally, the CBF was significantly higher at the infarct site of *Tia1*^Cx3cr1^-CKO mice on the 3^rd^ day after IS (Figure 1H-I). Collectively, these results suggested that TIA1 was significantly upregulated in microglia during acute phase of IS, and *Tia1* knockout in microglia alleviated the motor behavioral deficits in mice after IS.

### IS injury and the loss of neurons were alleviated in *Tia1*^Cx3cr1^-CKO mice after IS

**Figure 2:**
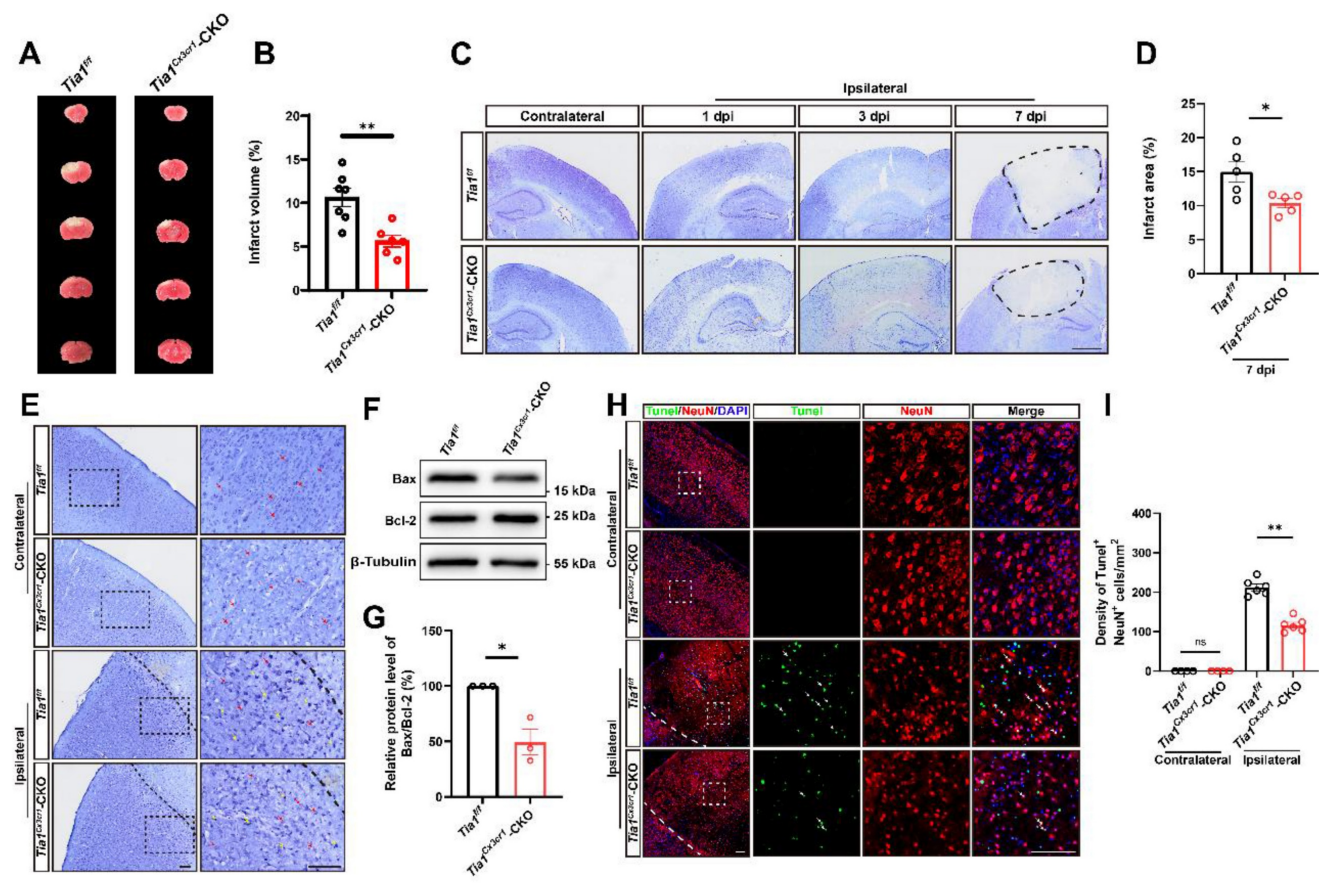
***Tia1* deficiency in microglia alleviated IS injury and neuronal loss in mice after IS.** (**A**) Representative images of TTC staining of *Tia1*^f/f^ and* Tia1*^Cx3cr1^-CKO mice on the 3^rd^ day after IS. (**B**) Quantitative analysis of the infarct volume of *Tia1*^f/f^ and* Tia1*^Cx3cr1^-CKO mice in TTC staining on the 3^rd^ day after IS as shown in (**A**) (n = 7 for the *Tia1*^f/f^ *mice*, and n = 6 for the *Tia1*^Cx3cr1^-CKO mice). (**C**) Representative images of Nissl staining in contralateral and infarct area of *Tia1*^f/f^ and* Tia1*^Cx3cr1^-CKO mice on the 1^st^, 3^rd^ and 7^th^ day after IS. Scale bars, 1 mm. (**D**) Quantitative analysis of the infarct area of *Tia1*^f/f^ and* Tia1*^Cx3cr1^-CKO mice by Nissl staining on the 7^th^ day after IS as shown in (**C**) (n = 5 mice per group). (**E**) Representative images of Nissl staining in the contralateral area and penumbra of *Tia1*^f/f^ and* Tia1*^Cx3cr1^-CKO mice on the 3^rd^ day after IS (Yellow arrow: shrunken Nissl bodies; red arrows: intact Nissl bodies). (**F**) Western blotting analysis of Bax and Bcl-2 expression in the infarct area of *Tia1*^f/f^ and* Tia1*^Cx3cr1^-CKO on the 3^rd^ day after IS. (**G**) Quantification of relative Bax/Bcl-2 expression level as shown in (**F**) (n = 3 mice per group, normalized to *Tia1*^f/f^ control mice). (**H**) Immunostaining analysis of TUNEL (green) and NeuN (red) in contralateral and infarct area of *Tia1*^f/f^ and* Tia1*^Cx3cr1^-CKO mice on the 3^rd^ day after IS. (**I**) Quantitative analysis of the density of TUNEL^+^/NeuN^+^ cells as shown in (**H**) (n = 4 mice in the contralateral area and n = 6 mice in the infarct area per group). Scale bars, 100 µm. Data were shown as mean ± s.e.m. *ns = no significance*, ^*^P < 0.05,^ **^P < 0.01, compared with *Tia1*^f/f^ mice or control.

We next examined the loss of neurons in the infarct core in these mice on the 3^rd^ day after IS. As expected, TTC and Nissl staining showed a significant reduction in the infarct area in *Tia1*^Cx3cr1^-CKO mice after IS, indicating that *Tia1* knockout in microglia alleviated IS injury (Figure 2A-D). Nissl staining is utilized to examine Nissl bodies in Nissl^+^ cells for determining neuronal integrity. As shown in Figure 2E, fewer shrunken (yellow arrows) and more intact (red arrows) Nissl bodies were found in *Tia1*^Cx3cr1^-CKO mice, suggesting less neuronal loss in *Tia1*^Cx3cr1^-CKO mice after IS. Moreover, the ratio of apoptosis-related protein Bax/Bcl-2 was lower in the infarct area of *Tia1*^Cx3cr1^-CKO mice (Figure 2F-G). Additionally, fewer TUNEL and NeuN double-positive signals were observed in *Tia1*^Cx3cr1^-CKO mice, indicating that reduction in neuronal loss was due to the decrease of neuronal apoptosis in *Tia1*^Cx3cr1^-CKO mice after IS (Figure 2H-I). Taken together, these results suggested that *Tia1* knockout in microglia attenuated IS injury and reduced neuronal loss in mice after IS.

### *Tia1* deficiency in microglia enhanced microglial activation, promoted polarization towards anti-inflammatory phenotypes, and alleviated inflammatory responses in mice after IS

**Figure 3:**
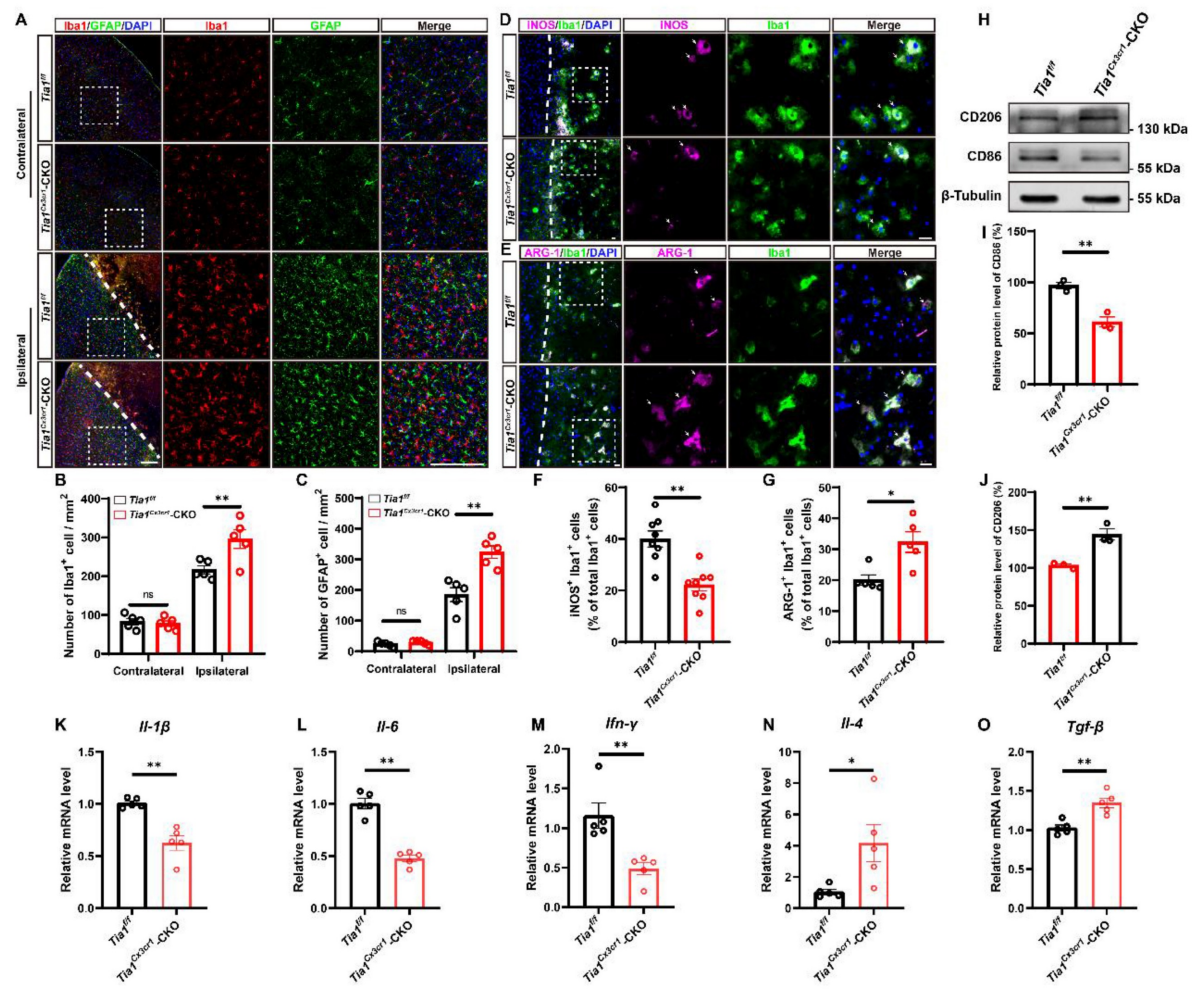
***Tia1* deficiency in microglia alleviated the inflammatory responses by regulating microglial polarization in mice after IS.** (**A**) Immunostaining analysis of Iba1 (red) and GFAP (green) in the contralateral area and penumbra of *Tia1*^f/f^ and* Tia1*^Cx3cr1^-CKO mice on the 3^rd^ day after IS. Scale bars, 200 µm. (**B**-**C**) Quantitative analysis of the density of Iba1^+^ (**B**) and GFAP^+^ (**C**) cells as shown in (**A**) (n = 5 mice per group). (**D**-**E**) Immunostaining analysis of iNOS (purple) (**D**) or ARG-1 (purple) (**E**) and Iba1 (green) in the infarct area of *Tia1*^f/f^ and* Tia1*^Cx3cr1^-CKO mice on the 3^rd^ day after IS. Scale bars, 20 µm. (**F**-**G**) Quantitative analysis of the percentages of iNOS^+^ (**F**, n = 8 per group) or ARG-1^+^ (**G**, n = 5 per group) cells in total Iba1^+^ cells as shown in (**D**) or (**E**), respectively. (**H**) Western blotting analysis of the expression of CD206 and CD86 in the infarct area of *Tia1*^f/f^ and* Tia1*^Cx3cr1^-CKO mice on the 3^rd^ day after IS. (**I**-**J**) Quantitative analysis of the relative expression of CD86 (**I**) and CD206 (**J**) as shown in (**H**) (normalized to the mean expression of control group, n = 3 per group). (**K**-**O**) Quantitative analysis of the relative mRNA levels of *Il-1β* (**K**), *Il-6* (**L**), *Ifn-γ* (**M**), *Il-4* (**N**), *Tgf-β* (**O**) in the infarct area of *Tia1*^f/f^ and* Tia1*^Cx3cr1^-CKO mice on the 3^rd^ day after IS (normalized to the mean expression of control group, n = 5 per group). Data were shown as mean ± s.e.m. ^*^P < 0.05, ^**^P < 0.01, compared with *Tia1*^f/f^ mice.

As previously reported, microglial activation significantly influences the progression of IS 36-38. Thus, we next examined whether the deletion of *Tia1* affected microglial responses to IS injury. As expected, more microglia were indeed activated around the lesion site in *Tia1*^Cx3cr1^-CKO mice on the 3^rd^ day after IS, whereas no significant difference was found in the contralateral uninjured brain regions (Figure 3A-B). Moreover, we found a significant increase in the number of activated astrocytes in *Tia1*^Cx3cr1^-CKO mice (Figure 3A, C), potentially attributable to the immune crosstalk between microglia and astrocytes after IS 39, 40.

As previously discussed, microglia exhibited both conventionally activated and alternatively activated functions during IS injury, characterized by distinct gene expression profiles in each polarization state. To investigate the effects of *Tia1* deletion on microglial function, we detected the proportions of conventionally activated (pro-inflammatory responses) and alternatively activated microglia (anti-inflammatory responses) in *Tia1*^f/f^ and *Tia1*^Cx3cr1^-CKO mice via immunostaining of polarization markers on the 3^rd^ day after IS. As shown in Figure 3D-G, *Tia1*^Cx3cr1^-CKO mice exhibited a significant reduction in the number of iNOS^+^ microglia (conventionally activated microglial marker) in the infarct area, accompanied by a notable increase in the percentage of ARG-1^+^ microglia (alternatively activated microglial marker). In addition, we found that the expression of the conventionally activated microglial marker CD86 was significantly decreased, whereas the alternatively activated microglial marker CD206 was markedly increased in* Tia1*^Cx3cr1^-CKO mice after IS (Figure 3H-J). Collectively, these results indicated that *Tia1* deletion promoted a shift in microglial polarization toward an anti-inflammatory phenotype after IS.

Furthermore, quantification of inflammatory cytokines in the infarct tissue revealed that the expression levels of pro-inflammatory factors, including interleukin-1β (*Il-1β*), interleukin-6 (*Il-6*) and interferon-γ (*Ifn-γ*) mRNA were significantly downregulated in *Tia1*^Cx3cr1^-CKO mice. Conversely, the expression levels of anti-inflammatory factors, such as interleukin-4 (*Il-4*) and transforming growth factor-β (*Tgf-β*) mRNA, were significantly upregulated in *Tia1*^Cx3cr1^-CKO mice after IS (Figure 3K-O), indicating that *Tia1* deletion in microglia alleviated the neuroinflammatory response in ischemic tissues after IS.

In conclusion, the above results suggested that the deletion of *Tia1* in microglia promoted the polarization of microglia towards an anti-inflammatory phenotype, alleviating the inflammatory response triggered by IS injury, which might contribute to the alleviation of brain injury in mice after IS.

### *Tia1* deficiency in microglia enhanced microglial phagocytosis in mice after IS

**Figure 4:**
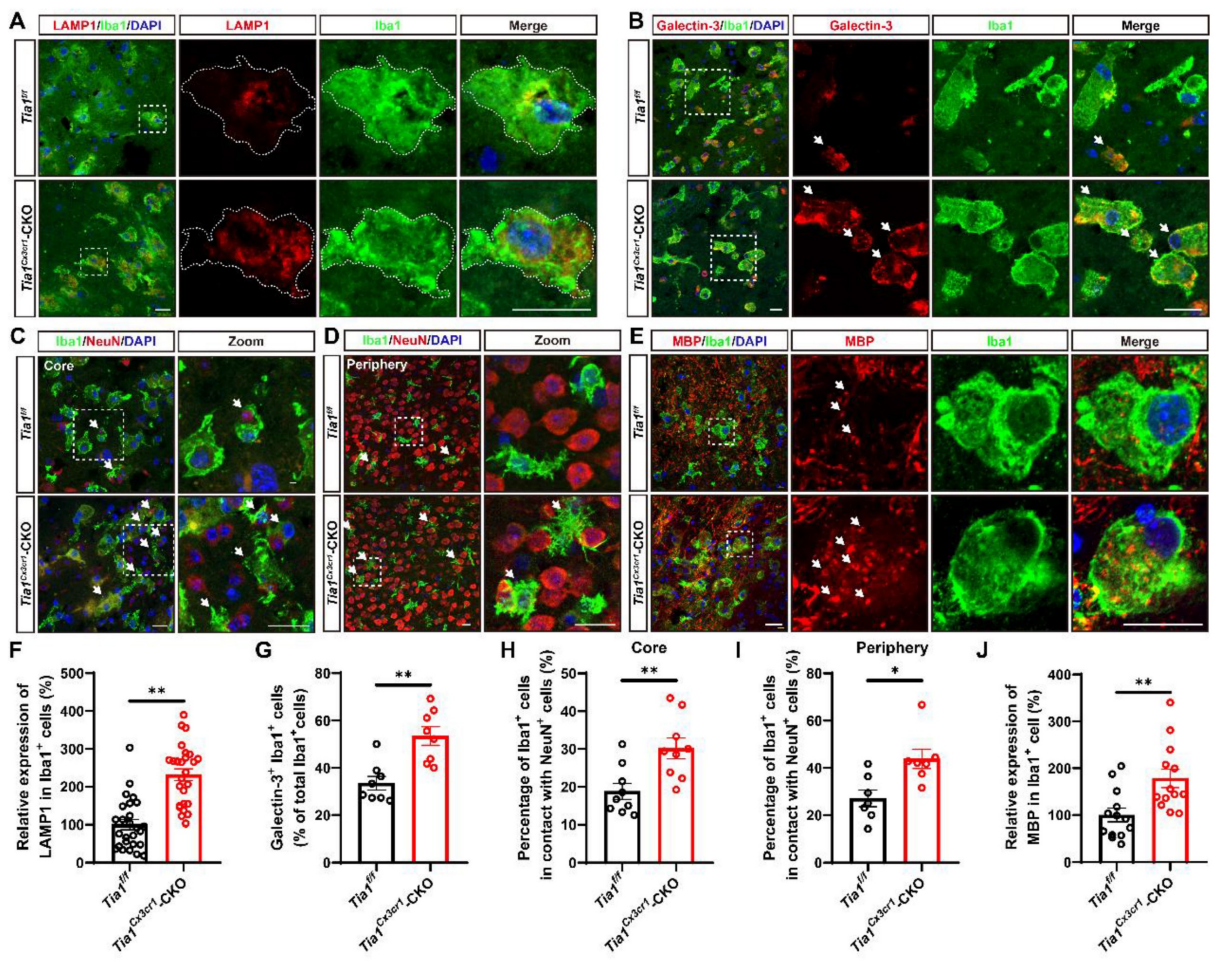
***Tia1* deficiency in microglia promoted the microglial phagocytosis in mice after IS.** (**A**-**B**) Immunostaining analysis of LAMP1 (red) (**A**) or Galectin-3 (red) (**B**) and Iba1 (green) in the infarct area of *Tia1*^f/f^ and* Tia1*^Cx3cr1^-CKO mice on the 3^rd^ day after IS. (**C**-**D**) Immunostaining analysis of Iba1 (green) and NeuN (red) in the infarct area (**C**) and penumbra (**D**) of *Tia1*^f/f^ and* Tia1*^Cx3cr1^-CKO mice on the 3^rd^ day after IS. (**E**) Immunostaining analysis of MBP (red) and Iba1 (green) in the infarct area of *Tia1*^f/f^ and* Tia1*^Cx3cr1^-CKO mice on the 3^rd^ day after IS. (**F**) Quantitative analysis of the expression of LAMP1 in Iba1^+^ cells as shown in (**A**) (n = 26 per group). (**G**) Quantitative analysis of the percentages of Galectin-3^+^ in total Iba1^+^ cells as shown in (**B**) (n = 8 per group). (**H**-**I**) Quantitative analysis of the percentages of Iba1^+^ cells in contact with NeuN^+^ cells in total Iba1^+^ cells in infarct area (**H**, n = 9 sections from 3 mice per group) and penumbra (**I**, n = 7 sections from 3 mice per group) as shown in (**C**-**D**). (**J**) Quantitative analysis of relative expression of MBP in Iba1^+^ cells as shown in (**E**) (normalized to the mean expression of control group, n = 13 per group). Scale bar, 20 µm. Data were shown as mean ± s.e.m. ^*^P < 0.05, ^**^P < 0.01, compared with *Tia1*^f/f^ mice.

Besides modulating the inflammatory response, microglia act as “battlefield sweeper” during the early phase of IS. By clearing cellular debris, they prevent the release of toxic contents from necrotic or damaged cells, thereby alleviating brain atrophy and motor dysfunction in the late stage of IS to some degree 41-43. To determine whether *Tia1* deletion affected microglial phagocytic activity, we performed immunofluorescence staining of brain tissue on the 3^rd^ day after IS in *Tia1*^f/f^ and *Tia1*^Cx3cr1^-CKO mice. As shown in Figure 4A and 4F, microglia in the infarct core of *Tia1*^Cx3cr1^-CKO mice exhibited higher expression levels of LAMP1 (a lysosomal marker). Moreover, the number of Galectin-3^+^ (a phagocytic marker) microglia was significantly increased in *Tia1*^Cx3cr1^-CKO mice (Figure 4B, G). Furthermore, we examined the localization relationship between microglia, neurons, and myelin within the infarct region. Notably, a significant increased number of microglia encapsulating NeuN^+^ signals were observed in the infarct area of *Tia1*^Cx3cr1^-CKO mice (Figure 4C, H). This phenomenon was also apparent in the penumbra surrounding the infarct core (Figure 4D, I). In addition, more MBP^+^ myelin debris were internalized by microglia in the infarct core of *Tia1*^Cx3cr1^-CKO mice (Figure 4E, J). Collectively, these results suggested that *Tia1* deletion in microglia enhanced the phagocytic activity of microglia and accelerated their clearance of cellular debris to alleviate IS injury.

### TIA1 promoted the shift toward pro-inflammatory phenotypes to promote neuronal death and inhibited the phagocytic ability of microglia *in vitro* after OGD

**Figure 5:**
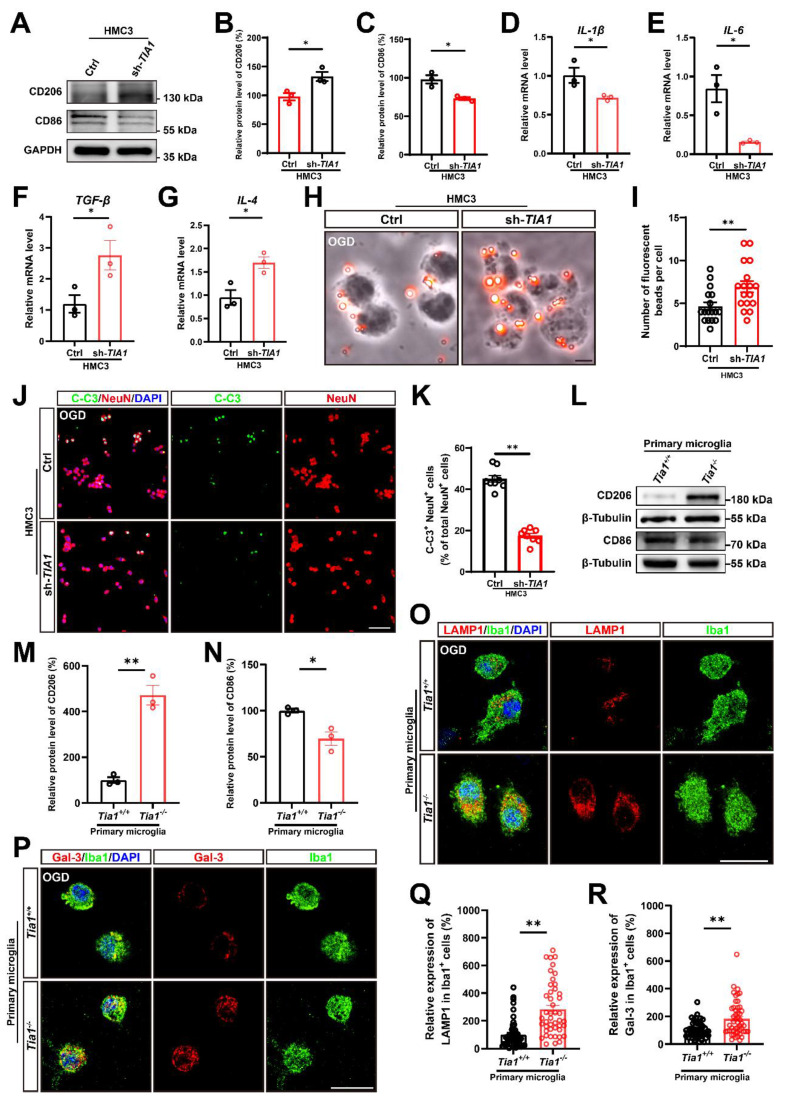
***Tia1* deficiency promoted microglial polarization and phagocytic function, alleviated inflammatory responses, and inhibited neuronal death after OGD.** (**A**) Western blotting analysis of the expression of CD206 and CD86 in control or *TIA1*-knockdown HMC3 cells after OGD. (**B**-**C**) Quantitative analysis of the relative expression of CD206 (**B**) and CD86 (**C**) as shown in (**A**) (normalized to the mean expression of control group, n = 3 per group). (**D**-**G**) Quantitative analysis of the relative mRNA levels of *Il-1β* (**D**), *Il-6* (**E**),* Tgf-β* (**F**) and* Il-4* (**G**) in control and *TIA1*-knockdown HMC3 cells after OGD (n = 3 per group). (**H**) Representative images of phagocytosis analysis after OGD in control and *TIA1*-knockdown HMC3 cells. Scale bars, 20 µm. (**I**) Quantitative analysis of the number of fluorescent beads per cell as shown in (**H**) (n = 18 per group). (**J**) Immunostaining analysis of NeuN (red) and C-C3 (green) of N2a cells after cocultured with control and* TIA1*-knockdown HMC3 cells after OGD. (**K**) Quantitative analysis of the percentages of C-C3^+^ cells among NeuN^+^ cells as shown in (**J**) (n = 9 in the control groups and n = 8 in the sh-*TIA1* group). Scale bars, 100 µm. (**L**) Western blotting analysis of the expression of CD206 and CD86 in primary cultured *Tia1*^+/+^ and *Tia1*^-/-^ microglia after OGD. (**M-N**) Quantitative analysis of relative CD206 (**M**) and CD86 (**N**) as shown in (**L**) (normalized to the mean expression of *Tia1*^+/+^ group, n = 3 per group). (**O-P**) Immunostaining analysis of LAMP1 (**O**) and Galectin-3 (**P**) (red), Iba1 (green) in primary cultured *Tia1*^+/+^ and *Tia1*^-/-^ microglia after OGD. Scale bars, 20 µm. (**Q**) Quantitative analysis of relative LAMP1 expression in Iba1^+^ cells as shown in (**O**) (n = 45 cells from 5 independent cell coverslips per group). (**R**) Quantitative analysis of relative Galectin-3 expression in Iba1^+^ cells as shown in (**P**) (n = 50 cells from 5 independent cell coverslips per group). Data were shown as mean ± s.e.m. ^*^P < 0.05, ^**^P < 0.01, compared with the control or *Tia1*^+/+^ group.

To further demonstrate the function and underlying mechanism of TIA1 in microglia after IS, we utilized the microglia cell line (HMC3 cells) to establish an OGD model *in vitro*. As expected, TIA1 was also significantly upregulated in HMC3 cells after OGD (Supplementary Figure S3A, F), indicating that TIA1 was involved in the microglial responses to hypoxia and glucose deprivation *in vitro*. To delve deeper into this phenomenon, a sh-*TIA1* plasmid was constructed and transfected into HMC3 cells to generate a stable *TIA1*-knockdown HMC3 cell line for subsequent experiments (Supplementary Figure S3B, C, G). Interestingly, in line with the *in vivo* findings, *TIA1* knockdown significantly induced the alternatively activated polarization of microglia, as evidenced by the increased expression of CD206 and the decreased expression of CD86 (Figure 5A-C). Meanwhile, we found that *TIA1-*knockdown significantly inhibited the expression of pro-inflammatory factors (*Il-1β* and *Il-6* mRNA), while promoting the expression of anti-inflammatory factors (*Tgf-β and Il-4* mRNA) (Figure 5D-G) in HMC3 cells 4 h after OGD, which were consistent with the *in vivo* observations.

We also examined the role of TIA1 in microglial phagocytic activity *in vitro* after OGD. Using a fluorescent bead uptake assay, we found that *TIA1*-knockdown significantly enhanced the uptake of fluorescent beads by HMC3 cells 4 h after OGD. (Figure 5H, I). Additionally, to confirm whether *TIA1-*knockdown in microglia affected neuronal survival after OGD *in vitro*, we established a neuron-microglia co-culture system. As expected, the percentage of C-C3^+^ and NeuN^+^ N2a cells co-cultured with *TIA1*-knockdown-HMC3 cells was significantly decreased (Figure 5J, K), indicating that *TIA1*-knockdown in microglia promoted neuronal survival after OGD treatment.

Consistent with the findings in HMC3 cells, we found that in primary cultured microglia subjected to OGD, the protein level of CD206 was significantly increased, while the protein level of CD86 was obviously decreased in *Tia1^-/-^* microglia (Figure 5L-N), suggesting that *Tia1* deficiency might promote the anti-inflammatory function of primary cultured microglia. Furthermore, the expression of the LAMP1 was significantly increased in *Tia1*^-/-^ microglia (Figure 5O, Q). Likewise, the expression of Galectin-3 was also notably elevated in *Tia1*^-/-^ microglia (Figure 5P, R). These results indicated that *Tia1* deficiency might enhance the phagocytic ability in primary cultured microglia after OGD treatment.

To further confirm the role of TIA1 in microglia following OGD, *TIA1*-overexpressing HMC3 cells were established (Supplementary Figure S3D, E, H). Indeed, *TIA1*-overexpression promoted the polarization of HMC3 cells toward a pro-inflammatory phenotype, characterized by increased expression of pro-inflammatory cytokines and reduced levels of anti-inflammatory factors (Supplementary Figure S4A-G). In contrast to *TIA1*-knockdown HMC3 cells, *TIA1*-overexpression markedly decreased lysosomal marker expression in HMC3 cells and inhibited the phagocytic capacity after OGD (Supplementary Figure S4H, I, K, L). Furthermore, co-culturing with *TIA1*-overexpressing HMC3 cells following OGD led to a significant increase in the apoptotic percentages of N2a neurons, as indicated by elevated Cleaved-caspase3^+^/NeuN^+^ cells (Supplementary Figure S4J, M).

Taken together, these *in vitro* results suggested that TIA1 promoted the shift toward pro-inflammatory phenotypes to promote neuronal death and inhibited the phagocytic ability of microglia *in vitro* after OGD.

### TIA1 served as a critical regulator of SGs formation in microglia after IS or OGD

**Figure 6:**
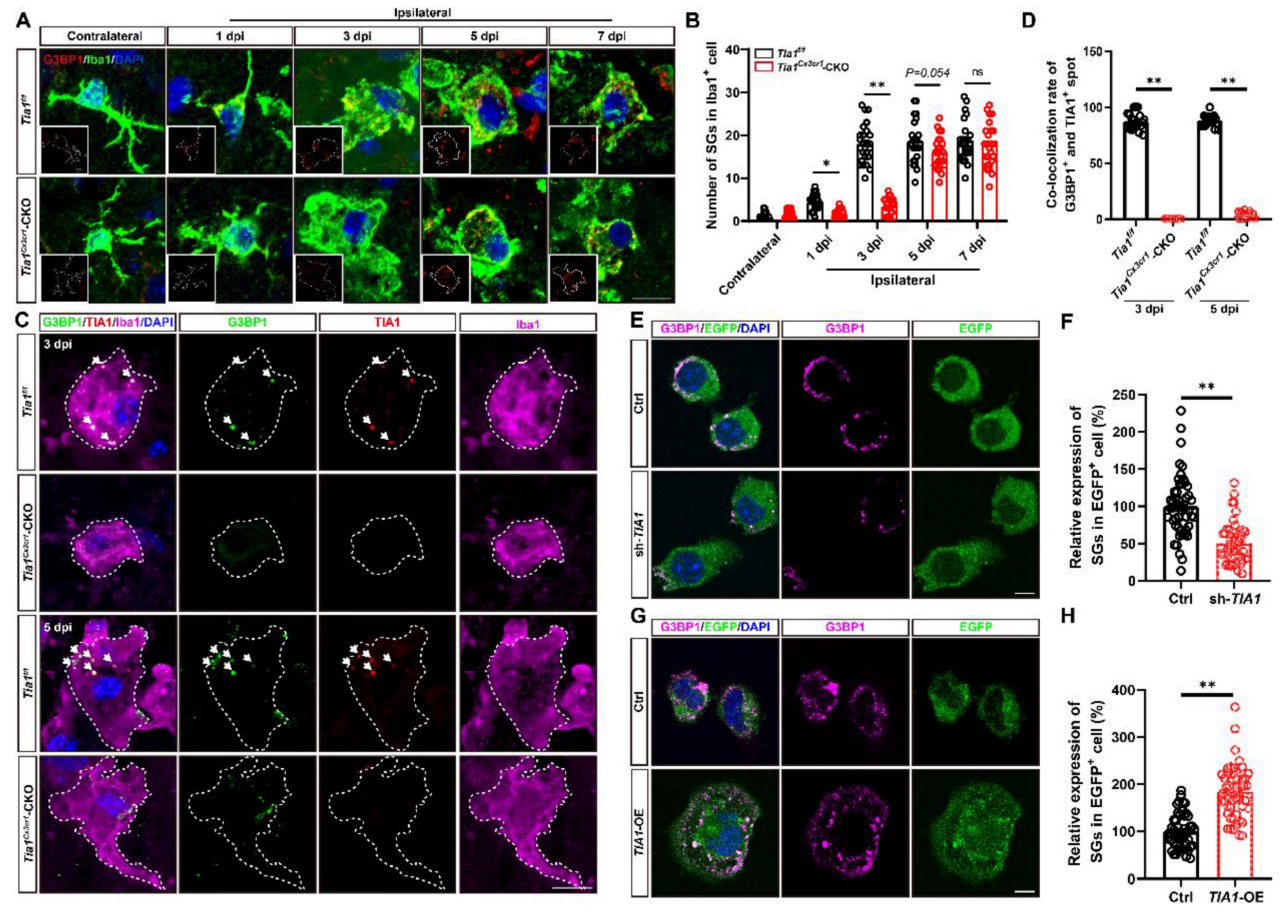
***Tia1* deficiency in microglia impaired the formation of SGs in mice after IS.** (**A**) Immunostaining analysis of G3BP1 (red) and Iba1 (green) in contralateral and infarct area of *Tia1*^f/f^ and *Tia1*^Cx3cr1^-CKO mice on the 1^st^, 3^rd^, 5^th^ and 7^th^ day after IS. (**B**) Quantitative analysis of the number of SGs in Iba1^+^ cells as shown in (**A**) (n = 25 cells from 5 mice). (**C**) Immunostaining analysis of G3BP1 (green), TIA1 (red) and Iba1 (purple) in infarct area of *Tia1*^f/f^ and *Tia1*^Cx3cr1^-CKO mice on the 3^rd^ and 5^th^ day after IS. (**D**) Quantitative analysis of co-location percentages of G3BP1^+^ and TIA1^+^ spots in Iba1^+^ cells as shown in (**C**) (n = 20 per group). (**E, G**) Immunostaining analysis of G3BP1 (purple) in sh-*TIA1* (**E**) or *TIA1*-OE (**G**) HMC3 cells after OGD. (**F, H**) Quantitative analysis of the relative expression of G3BP1 as shown in (**E**) or (**G**), respectively (normalized to the mean expression of control group, n = 50 cells from 5 independent cell coverslips per group. Scale bars, 20 µm. Data were shown as mean ± s.e.m. *ns = no significance*, ^*^P < 0.05, ^**^P < 0.01, compared with control.

As previously mentioned, TIA1 functions as an RBP that binds to target mRNAs during cellular stress, facilitating the assembly of SGs 18, 21. Given the upregulation of TIA1 expression in microglia after IS, we hypothesized that the microglial TIA1 might regulate SGs formation. G3BP1, a marker of SGs was used to detect the expression of SGs in microglia in the infarct area. Under normal physiological conditions, SGs were not detectably expressed in microglia. However, during IS, G3BP1^+^ signals were significantly increased in microglia, peaking by the 3^rd^ day after IS, and remaining elevated thereafter (Figure 6A-B), suggesting a functional role for SGs in microglia during ischemic stress. Surprisingly, while *Tia1* deletion in microglia did not completely inhibit SGs formation, as a small number of SGs were still persisted in microglia of *Tia1*^Cx3cr1^-CKO mice on the 3^rd^ day after IS, indicating a TIA1-independent compensatory pathway for SGs formation. Triple immunostaining for TIA1, G3BP1 and Iba1 showed that in *Tia1*^f/f^ mice, the majority of TIA1^+^ signals were co-localized with G3BP1^+^ spots in microglia, indicating that TIA1 promoted the formation of most SGs in microglia after IS. Conversely, in *Tia1*^Cx3cr1^-CKO mice, although TIA1^+^ SGs foci were absent by day 5 post-IS, residual G3BP1^+^ SGs were still detected, confirming that microglial TIA1 deletion did not completely abrogate SGs formation (Figure 6C-D). These results suggested that the deletion of microglial *Tia1* impaired SGs formation in microglia, particularly delaying their assembly during the early phases of IS. Similar results were found *in vitro*, *TIA1*-knockdown in HMC3 cells partially reduced SGs formation, whereas *TIA1-*overexpression promoted the SGs formation after OGD (Figure 6E-H). Collectively, these results suggested that TIA1 served as a critical regulator of SGs formation in microglia after IS or OGD injury.

### IGF2 in microglia was upregulated through decreasing the sequestration of its mRNA into SGs in *Tia1*^Cx3cr1^-CKO mice after IS

**Figure 7:**
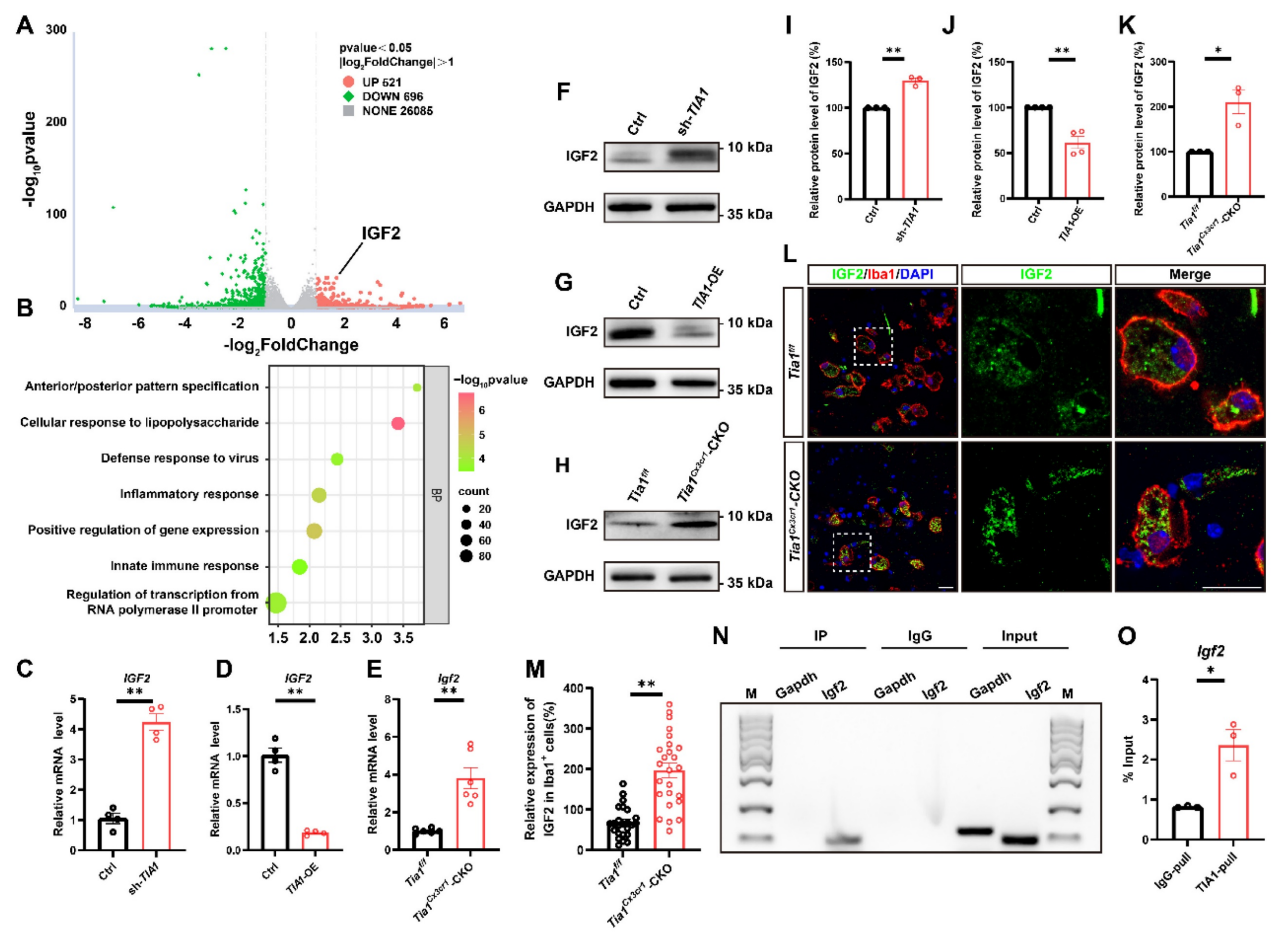
***Tia1* deficiency in microglia upregulated the expression of IGF2 in mice after IS.** (**A**) RNA-seq-based heatmap of differentially expressed mRNAs in the control and *TIA1*-knockdown HMC3 cells after OGD (n = 3 per group). (**B**) GO analysis of RNA-seq data in the control and *TIA1*-knockdown HMC3 cells after OGD (n = 3 per group). (**C**) Quantitative analysis of the relative mRNA levels of *Igf2* in control and *TIA1*-knockdown HMC3 cells after OGD (normalized to the mean expression of control group, n = 4 per group). (**D**) Quantitative analysis of the relative mRNA levels of *Igf2* in control and *TIA1*-overexpressing HMC3 cells after OGD (normalized to the mean expression of control group, n = 4 per group). (**E**) Quantitative analysis of the relative mRNA levels of *Igf2* on the 3^rd^ day after IS (normalized to the mean expression of control group, n = 6 per group). (**F**) Western blotting analysis of the expression of IGF2 in control and *TIA1*-knockdown HMC3 cells after OGD. (**G**) Western blotting analysis of expression of IGF2 in control and *TIA1*-overexpressing HMC3 cells after OGD. (**H**) Western blotting analysis of the expression of IGF2 in the infarct area of *Tia1*^f/f^ and *Tia1*^Cx3cr1^-CKO mice on the 3^rd^ day after IS. (**I**) Quantitative analysis of the relative expression of IGF2 as shown in (**F**) (normalized to the mean expression of control group, n = 3 per group). (**J**) Quantitative analysis of the expression of IGF2 as shown in (**G**) (normalized to the mean expression of control group, n = 3 per group). (**K**) Quantitative analysis of the relative expression of IGF2 as shown in (**H**) (normalized to the mean expression of control group, n = 3 per group). (**L**) Immunostaining analysis of Iba1 (red) and IGF2 (green) in the infarct area of *Tia1*^f/f^ and* Tia1*^Cx3cr1^-CKO mice on the 3^rd^ day after IS. Scale bar, 20 µm. (**M**) Quantitative analysis of relative expression of IGF2 in Iba1^+^ cells as shown in (**L**) (normalized to the mean expression of control group, n = 25 cells). (**N-O**) Agarose gel electrophoresis (**N**) and qRT-PCR (**O**) for the analysis of *IGF2* mRNA in mice by RIP assay (n = 3 per group). Data were shown as mean ± s.e.m. ^*^P < 0.05, ^**^P < 0.01, compared with control group.

To investigate whether TIA1-associated SGs regulate microglial activity by controlling specific gene expression, mRNA sequencing was performed in control and *TIA1*-knockdown HMC3 cells 4 h after OGD. As shown in Figure 7A, among differentially expressed genes, IGF2 was significantly upregulated in *TIA1*-knockdown HMC3 cells. GO analysis revealed that *TIA1*-knockdown significantly affected microglial immune function and inflammatory response pathways (Figure 7B), suggesting a potential association between TIA1-mediated SGs formation and IGF2 regulation in stressed microglia. IGF2, a pleiotropic polypeptide abundantly expressed in CNS, influences neurogenesis and neuroinflammation, with dysregulated expression linked to CNS disorders such as anxiety and Alzheimer's disease 44-46. Given that multiple RBPs recruited their target mRNAs into SGs to repress translation, we hypothesize that *Igf2* is a mRNA target of TIA1 in microglia, and knockout/knockdown of *Tia1* impairs its binding to *Igf2*, reducing *Igf2* recruitment into SGs. Indeed, *IGF2* mRNA levels were significantly elevated in *TIA1*-knockdown HMC3 cells (Figure 7C) and reduced in *TIA1*-overexpressing HMC3 cells after OGD (Figure 7D), respectively.

Interestingly, *Igf2* mRNA expression was also significantly upregulated in infarct tissues from *Tia1*^Cx3cr1^-CKO mice after IS (Figure 7E). Furthermore, IGF2 protein levels was also significantly upregulated in *TIA1*-knockdown HMC3 cells following OGD, whereas it was reduced in *TIA1*-overexpressing HMC3 cells (Figure 7F, G, I, J). Notably, IGF2 levels were significantly upregulated in infarct tissues of *Tia1*^Cx3cr1^-CKO mice (Figure 7H, K), and mainly in microglia of *Tia1*^Cx3cr1^-CKO mice on the 3^rd^ day after IS (Figure 7L-M). To investigate whether TIA1-mediated SGs sequester *Igf2* mRNA in microglial cells, RIP combined with qRT-PCR was performed. The results confirmed the binding of TIA1 protein to *Igf2* mRNA (Figure 7N-O). Taken together, these results suggested that microglial IGF2 upregulation in *Tia1*^Cx3cr1^-CKO mice was due to reduced sequestration of *Igf2* mRNA into TIA1-dependent SGs after IS.

### The alleviation of IS injury in *Tia1*^Cx3cr1^-CKO mice was through upregulation of IGF2 signaling

**Figure 8:**
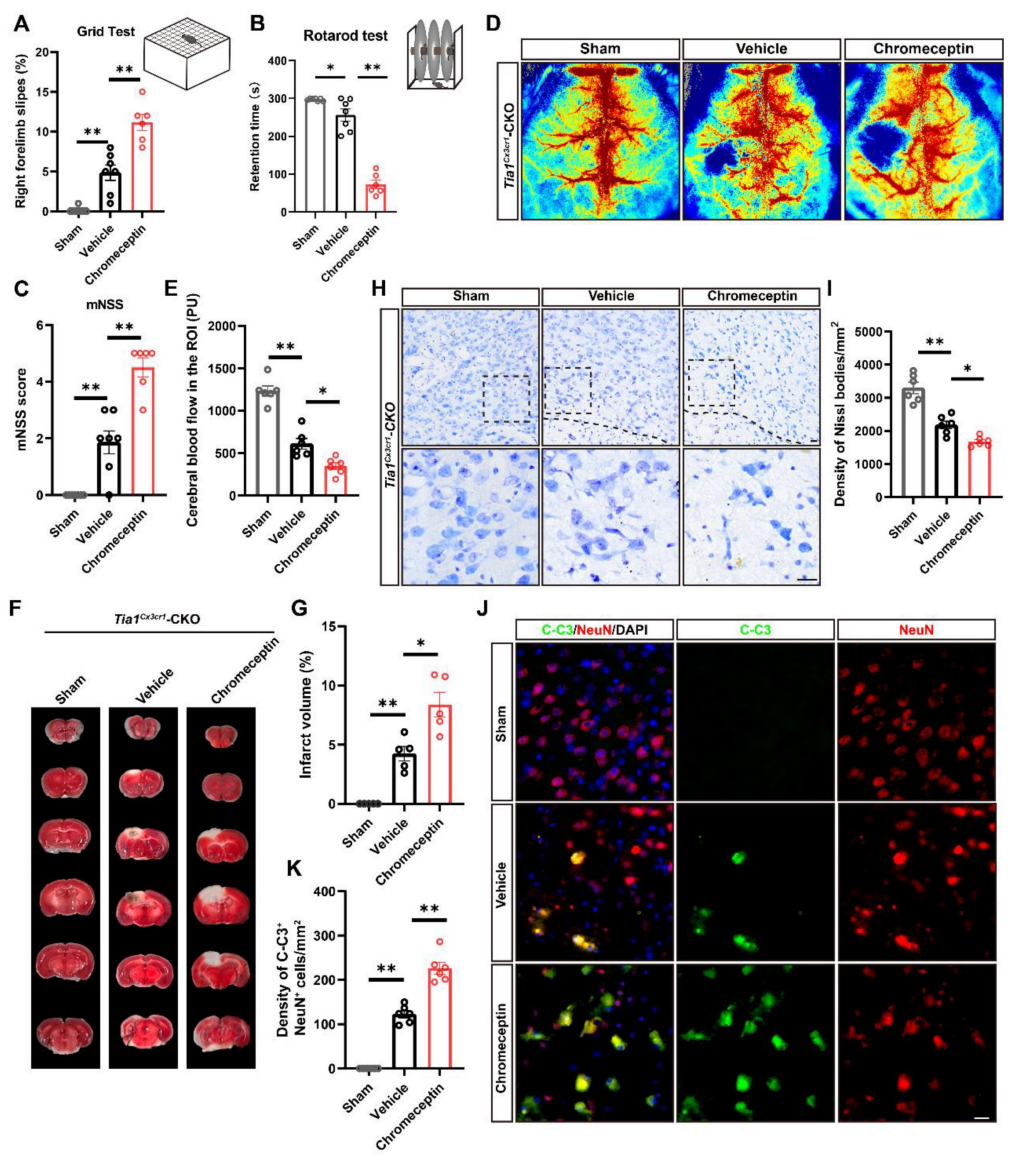
** Inhibition of microglial IGF2 signaling aggravated the motor behavioral deficits and neurological damage in* Tia1*^Cx3cr1^-CKO mice after IS.** (**A-B**) Behavioral analysis of *Tia1*^Cx3cr1^-CKO mice by grid test (**A**) and rotarod test (**B**) on the 3^rd^ day after chromeceptin treatment following IS injury (n = 6 mice per group for sham and chromeceptin, and n = 7 mice for the vehicle group). (**C**) Quantitative analysis of mNSS scores in *Tia1*^Cx3cr1^-CKO mice on the 3^rd^ day after chromeceptin treatment following IS injury (n = 6 mice per group for sham and chromeceptin, and n = 7 mice for the vehicle group). (**D**) Representative images of CBF changes by LSCI in *Tia1*^Cx3cr1^-CKO mice on the 3^rd^ day after chromeceptin treatment following IS injury. (**E**) Quantitative analysis of the CBF changes of *Tia1*^Cx3cr1^-CKO mice on the 3^rd^ day after chromeceptin treatment following IS injury as shown in (**D**) (n = 6 mice per group). (**F**) Representative images of TTC staining of *Tia1*^Cx3cr1^-CKO mice on the 3^rd^ day after chromeceptin treatment following IS injury. (**G**) Quantitative analysis of the infarct volume of *Tia1*^Cx3cr1^-CKO mice in TTC staining on the 3^rd^ day after chromeceptin treatment following IS injury as shown in (**F**) (n = 5 mice per group). (**H**) Representative images of Nissl staining in penumbra of *Tia1*^Cx3cr1^-CKO mice on the 3^rd^ day after chromeceptin treatment following IS injury. (**I**) Quantitative analysis of the density of Nissl bodies as shown in (**I**) (n = 6 mice per group). (**J**) Immunostaining analysis of C-C3 (green) and NeuN (red) in infarct area of *Tia1*^Cx3cr1^-CKO mice on the 3^rd^ day after chromeceptin treatment following IS injury. (**K**) Quantitative analysis of the density of C-C3^+^/NeuN^+^ cells as shown in (**J**) (n = 6 mice per group). Scale bars, 20 µm. Data were shown as mean ± s.e.m. ^*^P < 0.05, ^**^P < 0.01, compared with control group.

To investigate the relationship between IGF2 signaling and the alleviation of IS injury in *Tia1*^Cx3cr1^-CKO mice, chromeceptin (an IGF2 inhibitor) was administered to mice 47, 48. Mice received daily intraperitoneal injections of chromeceptin (1 mg/kg) starting 3 days prior to IS induction, and IGF2 expression in microglia was assessed on the 3^rd^ day after IS (Figure S5A-B). Subsequent behavioral analyses including grid walk, rotarod and mNSS tests revealed that chromeceptin treatment significantly blocked the alleviation of the motor behavioral deficits observed in *Tia1*^Cx3cr1^-CKO mice after IS (Figure 8A-C). Furthermore, LSCI indicated that chromeceptin also significantly blocked the alleviation CBF damage at the lesion site in *Tia1*^Cx3cr1^-CKO mice on the 3^rd^ day after IS (Figure 8D-E). TTC staining further revealed larger infarct volumes in chromeceptin-treated* Tia1*^Cx3cr1^-CKO mice compared to controls (Figure 8F, G). Chromeceptin also abolished the reduction in neuronal loss and apoptosis in *Tia1*^Cx3cr1^-CKO mice after IS (Figure 8H-K). These results suggested that the alleviation of the motor behavioral deficits and neuroprotection in *Tia1*^Cx3cr1^-CKO mice were partially mediated by IGF2-dependent signaling pathways, as evidenced by the reversal of beneficial effects following IGF2 inhibition.

**Figure 9:**
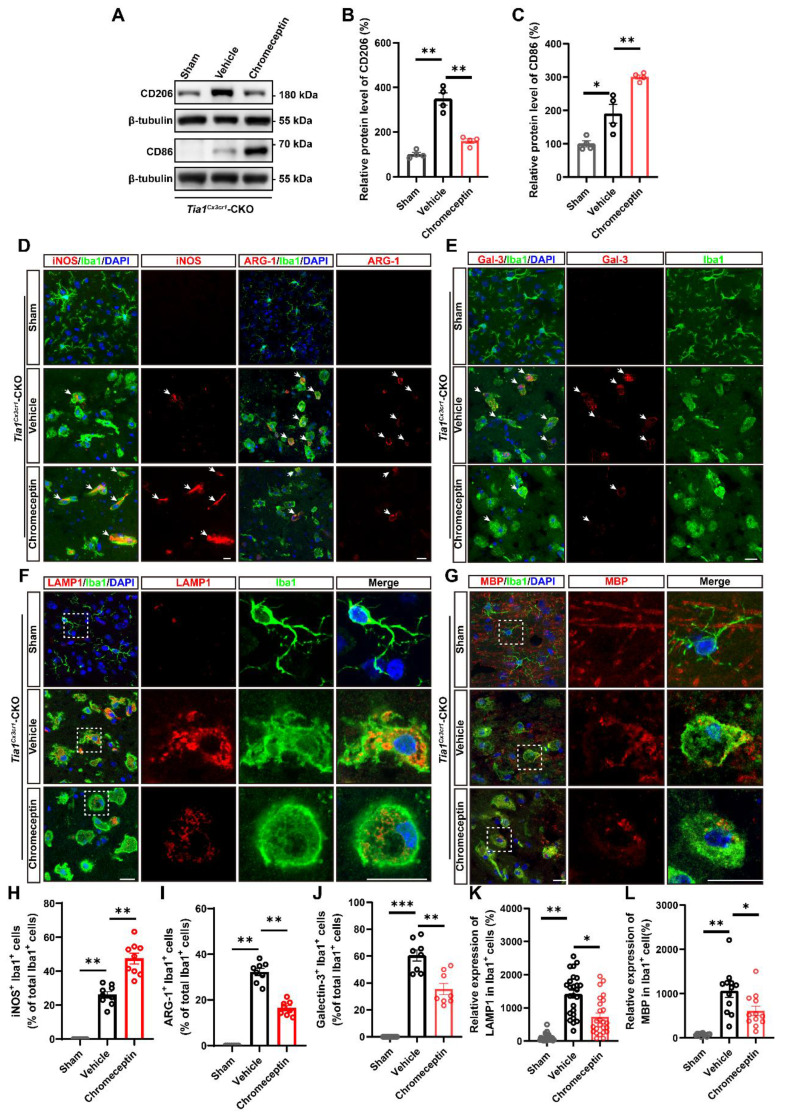
** Inhibition of IGF2 impaired microglial phagocytic activity and aggravated neuroinflammatory responses in *Tia1*^Cx3cr1^-CKO mice after IS.** (**A**) Western blotting analysis of the expression of CD206 and CD86 in the infarct area of *Tia1*^Cx3cr1^-CKO mice on the 3^rd^ day after chromeceptin treatment following IS injury. (**B**-**C**) Quantitative analysis of the relative expression of CD206 (**B**) and CD86 (**C**) as shown in (**A**) (normalized to the mean expression of control group, n = 4 per group). (**D-G**) Immunostaining analysis of iNOS (red) or ARG-1 (red) and Iba1 (green) (**D**), Galectin-3 (red) and Iba1 (green) (**E**), LAMP1 (red) and Iba1 (green) (**F**), MBP (red) and Iba1 (green) (**G**) in the infarct area of *Tia1*^Cx3cr1^-CKO mice on the 3^rd^ day after chromeceptin treatment following IS injury. (**H-I**) Quantitative analysis of the percentages of iNOS^+^ (**H**, n = 9 sections from 3 mice per group) or ARG-1^+^ (**I**, n = 8 sections from 3 mice per group) cells in total Iba1^+^ cells as shown in (**D**). (**J**) Quantitative analysis of the relative expression of Galectin-3 as shown in (**E**) (normalized to the mean expression of sham group, n = 8 per group). (**K**) Quantitative analysis of the relative expression of LAMP1 as shown in (**F**) (normalized to the mean expression of sham group, n = 25 cells). (**L**) Quantitative analysis of the relative expression of MBP as shown in (**G**) (normalized to the mean expression of sham group, n = 12 per group). Scale bars, 20 µm. Data were shown as mean ± s.e.m. ^*^P < 0.05, ^**^P < 0.01, ^***^P < 0.001, compared with Sham or Vehicle group.

To assess whether IGF2 signaling regulates microglial dynamics in the post-ischemic brain, microglial activation states were analyzed in *Tia1*^Cx3cr1^-CKO mice following pharmacological IGF2 inhibition. The upregulation of CD86 and downregulation of CD206 expression were observed in the infarcted regions of chromeceptin-treated *Tia1*^Cx3cr1^-CKO mice (Figure 9A-C). Immunofluorescence staining further revealed a substantial increase in iNOS^+^ microglia and a concomitant reduction in ARG-1^+^ microglia percentages following chromeceptin treatment, indicating a shift toward conventionally activated microglial polarization upon IGF2 inhibition (Figure 9D, H, I). Quantitative analysis of inflammatory cytokines in infarct tissues confirmed the upregulated levels of pro-inflammatory cytokines and downregulated anti-inflammatory factors in chromeceptin-treated *Tia1*^Cx3cr1^-CKO mice after IS (Figure S5C-G). Additionally, a significant decrease of LAMP1 expression was observed in microglia of chromeceptin-treated *Tia1*^Cx3cr1^-CKO mice, suggesting a reduction of microglial phagocytic function (Figure 9F, K). Similarly, Galectin-3/Iba1 co-staining demonstrated diminished Galectin-3 expression in these microglia (Figure 9E, J). To corroborate these findings, myelin debris clearance by microglia was evaluated in infarcted regions. Chromeceptin-treated *Tia1*^Cx3cr1^-CKO mice exhibited significantly reduced myelin basic protein (MBP) debris accumulation within microglia compared to controls (Figure 9G, L).

To further confirm these observations at the *in vivo* level, we performed complementary *in vitro* experiments using primary cultured *Tia1*^+/+^ microglia. As shown in the Western blotting results, compared with the OGD group, IGF2 treatment significantly increased the expression of the alternatively activated marker CD206 while decreasing the classical activated marker CD86 in primary cultured microglia (Figure S6A-C). Moreover, immunofluorescence staining demonstrated that IGF2 treatment remarkably enhanced the expression of LAMP1 in Iba1^+^ microglia under OGD conditions (Figure S6D-E).

Collectively, these results suggested that IS injury in *Tia1*^Cx3cr1^-CKO mice was alleviated through upregulation of IGF2 signaling, as evidenced by aggravating motor behavioral deficits, decreasing cerebral hypoperfusion and aggravating neuronal injury by inhibition of IGF2 signaling. Mechanistically, IGF2 inhibition promoted microglial polarization toward a conventionally activated phenotype, suppressed phagocytic activity (via LAMP1 and Galectin-3 modulation), and amplified neuroinflammatory responses, ultimately aggravated the motor behavioral deficits in *Tia1*^Cx3cr1^-CKO mice after IS.

## Discussion

**Figure 10:**
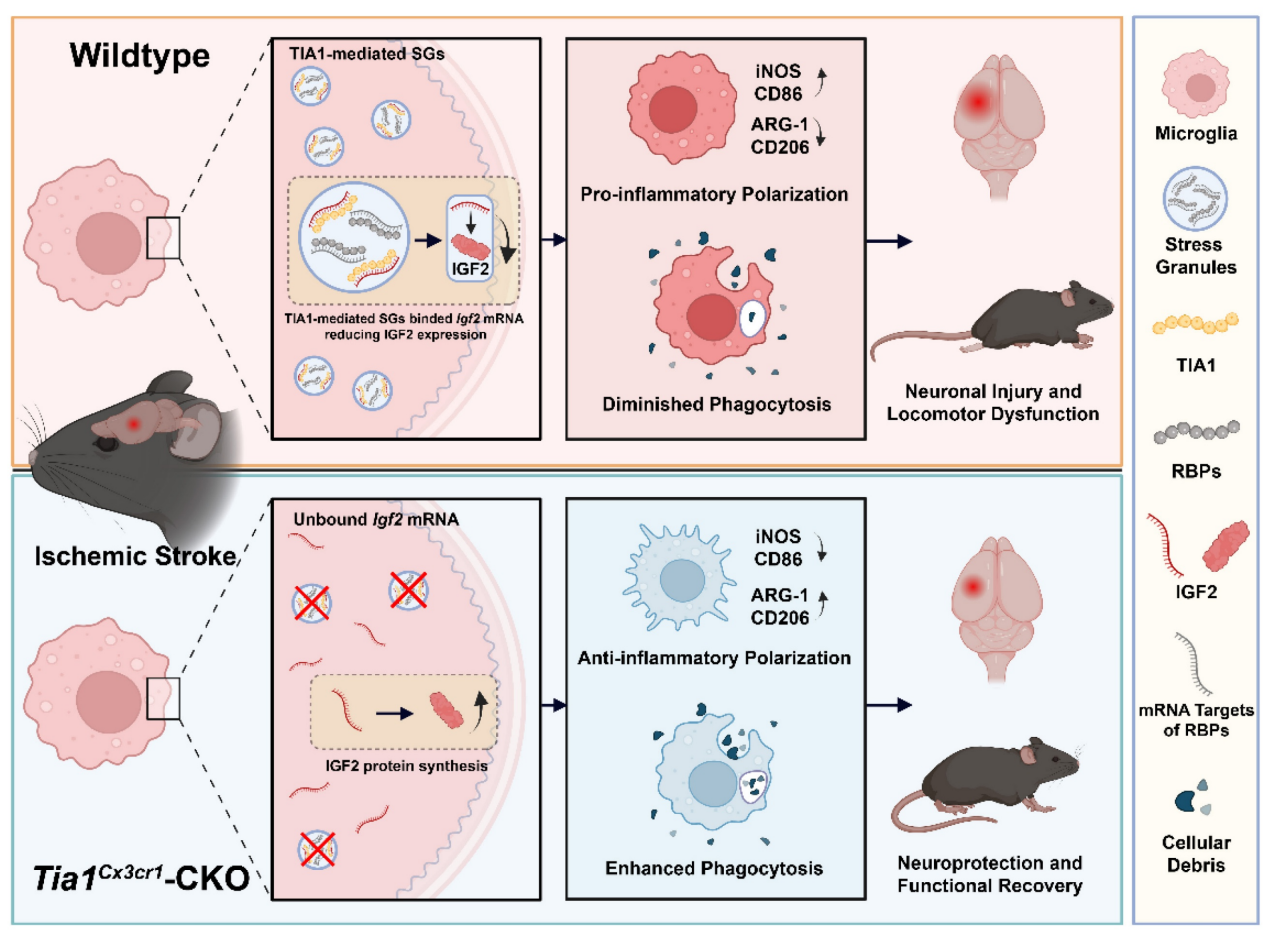
** The working model of microglial TIA1 in mice after IS.** TIA1 deletion in microglia impairs SGs formation and upregulated IGF2 expression through decreasing the sequestration of *Igf2* mRNA into SGs, which regulates microglial activation, anti-inflammatory polarization and phagocytic activity of microglia, ultimately alleviating neuron injury and accelerating behavioral function recovery in mice after IS.

This study elucidated the regulatory role of microglial TIA1 in IS progression and its underlying mechanisms. Conditional knockout of *Tia1* in microglia significantly alleviated the motor behavioral deficits by promoting alternatively activated polarization and enhancing the phagocytic activity of microglia, thereby attenuating neuroinflammation in mice after IS. Mechanistic investigations revealed that *Tia1* ablation partially disrupted the SGs formation in microglia, subsequently inducing upregulation of IGF2. The IGF2-mediated pathway exerted neuroprotective effects and alleviated the motor behavioral deficits by suppressing neuroinflammatory cascades after IS. However, pharmacological inhibition of IGF2 reversed these neuroprotective effects, as evidenced by impaired microglial phagocytosis, reactivated neuroinflammation, and aggravated the motor behavioral deficits in chromeceptin-treated* Tia1*^Cx3cr1^-CKO mice. Collectively, our findings identify the TIA1-IGF2 signaling as a critical regulator of microglia homeostasis and sustain pro-inflammatory response, orchestrating neuroinflammatory modulation and determining functional outcomes in mice after IS (Figure 10).

The pathological mechanism underlying IS manifests as a dynamic cascade of events, particularly during the acute and subacute phases, when cellular and molecular changes occur rapidly 49. In contrast, the chronic phase demonstrates a distinct biological profile where endogenous neuroplasticity mechanisms attain a biological plateau that substantially diminishes the therapeutic potential of exogenous interventions. At this recovery stage, neurorehabilitation strategies emphasizing activity-dependent cortical reorganization, such as constraint-induced movement therapy and task-specific training, assume paramount importance for optimizing functional outcomes 50. Therefore, early intervention during the acute phase of IS is critical, as it can profoundly influence the disease trajectory.

Interestingly, our study found that *Tia1*^Cx3cr1^-CKO mice showed the behavioral recovery as early as day 1 after IS (Figure 1), while reduction in infarct size began day 3 after IS (Figure 2), suggesting a temporal difference between behavioral and morphological improvements. This discrepancy might be due in part to differences in the sensitivity of the assessment methods. Despite the sophistication of current technologies, their sensitivity thresholds remain a limitation. During the earliest disease stages, the pathological alterations fall below the detection limit of existing methodologies (e.g., Nissl and HE staining), evading identification. In contrast, subtle functional impairments such as a slight decline in the efficiency of specific neural networks can potentially be captured earlier through refined behavioral or cognitive assessments. Some literatures document this phenomenon across multiple neurodegenerative disorders, as well as psychiatric conditions 51, 52. In our animal model, some damaged/dying neurons were still involved in neural circuits after IS, and impairment of neural circuits could be detected by the behavioral assays, however, Nissl staining was used to detect the cerebral infarct area (with no capacity to detect the presence of damaged yet viable neurons). Thus, the behavioral phenotypes might precede the emergence of histological phenotypes.

During the early stage of IS, brain cells such as neurons, glial cells, and endothelial cells in the infarct core region suffer from OGD. Microglia respond rapidly to acute stroke stimulation. Damaged neurons and glial cells release damage-associated molecular patterns (DAMPs), which trigger microglial activation and initiate neuroinflammatory responses. Activated microglia quickly migrate to the lesion core, where they polarize into reactive states and play a pivotal role in regulating neuroinflammation, pathophysiological mechanism critically involved in IS progression 53-55. In this study, we found TIA1, a core element of SGs, was upregulated in microglia at early stage of IS, and the microglia-specific* Tia1* knockout promoted the neuroprotective effect in mice after IS. The dynamics of SGs mediated by RBPs are profoundly altered during the early phases of ischemia, mediating both cellular stress adaptation and neuronal fate determination 56. Previous studies reported TIA1 mediated SGs assembly reduces neuronal apoptosis and neuroinflammation 22, these investigations utilized global *Tia1* knockout models rather than cell-type-specific approaches, leaving unresolved the cell-type-specific regulatory mechanisms of microglial TIA1. Emerging evidence found that TIA1 reduction increased microglial phagocytosis in advanced tauopathy 57, while a recent study reported that CX3CR1 driven knockout of TIA1 in microglia greatly reduced microglial inflammation 58, aligning with our findings. Importantly, we reveal the potential mechanism of microglial TIA1 intervention in regulating microglia polarization and neuroinflammation during IS progression. This cell type-specific duality underscores the importance of spatial and temporal precision when evaluating RBPs functions in CNS pathologies.

Beyond regulating polarization and neuroinflammation, microglial TIA1 may also contribute to neuroprotection by modulating CBF, which is a key determinant of ischemic tissue survival. To rule out confounding factors in our assessment of CBF, we implemented strict baseline controls between *Tia1*^Cx3cr1^-CKO mice and *Tia1*^f/f^ mice: prior to modeling, the two groups were identical in terms of physiological status and housing environment. During the modeling phase, IS injury was induced in both groups using the same method. This injury inevitably led to a decrease in CBF perfusion in the ischemic core region due to vascular occlusion. However, post-modeling assessments revealed that the magnitude of CBF reduction was significantly higher in *Tia1*^Cx3cr1^-CKO mice compared to *Tia1*^f/f^ mice, suggesting that *Tia1* knockout in microglia protects ischemic brain tissue by modulating the pathological processes following ischemic injury.

The neurovascular unit (NVU) is consisting of endothelial cells, glial cells (astrocytes, microglia and other glial elements), mural cells (vascular smooth muscle cells and pericytes), perivascular macrophages and neurons 59. The wide array of signaling pathways between interacting NVU cells maintain the integrity of the blood-brain barrier (BBB), and the regulation of CBF for brain homeostasis. Recent studies have shown that diverse microglia-mediated vascular effects have been implicated in cerebral ischemia and other brain pathologies 59, 60. Microglial elimination triggers the capillary dilation and blood flow increases in mice 61. Microglia-derived TNF-α mediates the endothelial necroptosis aggravating BBB disruption after IS 62, and capillary-associated microglia regulate vascular structure and function through PANX1-P2RY12 coupling in mice 61. Consistent with these previous studies, we speculate that some factors released from capillary-associated *Tia1^-/-^* microglia promote the increase of CBF after IS. In the future study, we will identify the underlying molecular mechanism.

Although TIA1-associated mRNA targets have previously been identified in human embryonic stem cells (hESCs) and neurodevelopmental contexts under homeostatic conditions, its RNA-binding selectivity under stress remains poorly characterized 63. Through mRNA sequencing and GO analysis, we uncovered a stress-specific binding preference of microglial TIA1 for *Igf2* mRNA, orchestrating its sequestration into SGs following IS or OGD. Genetic ablation or knockdown of *Tia1* upregulates IGF2 expression in microglia, establishing a novel regulatory axis between TIA1 and IGF2 signaling. However, *Tia1* knockout/knockdown did not completely abolish the IGF2 recruitment to SGs, since other RBPs may also bind to *Igf2* through biochemical compensation mechanisms.

IGF2 is a single-chain polypeptide belonging to the insulin-related protein family, serves as a critical regulator of development and cancers 64-66. During early embryonic and fetal development, IGF2 is widely expressed in various somatic cells, however, its expression declines significantly after birth, persisting at high levels only in specialized tissues including the brain 67, 68. As an important immunomodulatory mediator, IGF2 confers anti-inflammatory properties to mature macrophages 69. Recent studies have shown that IGF2 alleviated experimental autoimmune encephalomyelitis (EAE) and dextran sodium sulfate (DSS)-induced colitis by programming macrophages to acquire an alternatively activated phenotype 70, 71. Treatment of MCAO rats with Eerdun Wurile significantly improves recovery from IS by upregulating the expression of IGF2 in microglia cells in the lesion, potentially prompting a conventionally activated polarization to alternatively activated polarization of microglia 72. Notably, IGF2 has been shown to alleviate neuroinflammation by inducing alternatively activated microglial polarization. It has been demonstrated that microglia are important expressers of IGF2 in the setting of CNS injury 73. Previous studies have further pointed out that IGF2 suppresses conventionally activated microglial polarization and reduces inflammatory factor production in LPS-treated mice, thereby relieving neuroinflammation 27. This post-transcriptional mechanism increases IGF2 expression 74, 75, thereby modulating its microglial function and alleviating neuroinflammation after IS injury.

Regarding the mechanistic association between impaired SGs formation and IGF2 upregulation, transcriptome sequencing previously performed demonstrated that *Igf2* mRNA was upregulated in TIA1-knockdown microglia following OGD. Additionally, results from RIP-qPCR demonstrated that TIA1 bound to *Igf2* mRNA (Figure 7N-O). These findings established a critical molecular link between “TIA1 deficiency” and the “subsequent upregulation of IGF2 observed in transcriptome data”. To further validate the specificity of the aforementioned binding relationship, bioinformatic analysis using the POSTAR3 database was supplemented in this study. As an authoritative platform dedicated to dissecting the coordinated post-transcriptional regulation mediated by RBPs 76, 77, retrieval and analysis of POSTAR3 confirmed that IGF2 is indeed a target mRNA of TIA1, which provided cross-validation evidence for the interaction between TIA1 and *Igf2* mRNA. Furthermore, results from functional validation experiments showed that significant differences in the polarization status and phagocytic capacity of microglia were observed following treatment with the IGF2 inhibitor chromeceptin or exogenous IGF2. Specifically, the IGF2 signaling pathway, as a downstream effector molecule of TIA1, was shown to regulate the polarization and phagocytic activity of microglia. While these preclinical insights demonstrate translational potential, further studies are warranted to validate the clinical relevance of the TIA1-IGF2 axis in human stroke patients and to determine the optimal therapeutic intervention.

Our study reveals that TIA1 regulates IGF2 expression through SGs, thereby affecting microglial function, but some questions need to be answered in the future. Firstly, the specific downstream signaling pathways of IGF2 that regulate microglial polarization and phagocytosis have not yet been explored. Secondly, whether TIA1 synergistically regulates microglial function by targeting other mRNA molecules in SGs (not just IGF2) requires further investigation. Thirdly, Cx3cr1-Cre allows for the knockout of genes in microglia and monocyte-derived macrophages, thus it is important to note that the phenotypes in *Tia1*^Cx3cr1^-CKO mice after IS may arise from the dual effects of TIA1-mediated SGs in both microglia and peripheral macrophages. Finally, the N2a cell line is used as an *in vitro* surrogate for primary neurons in this study. However, these immortalized cells exhibit molecular and functional differences with neurons and do not fully recapitulate primary neuronal physiology 78, which may influence the interpretation of microglia-neuron interactions. The inclusion of these limitations will provide directions for future research.

## Conclusion

This study reveals a novel molecular mechanism by which microglial TIA1 governs post-ischemic neuroinflammation and tissue repair. TIA1 modulates the microglia homeostasis to promote pro-inflammatory responses and inhibit the phagocytic clearance of cellular debris via SGs-mediated translational control of *Igf2* mRNA sequestration in mice after IS. Collectively, these findings position the microglial TIA1-IGF2 signaling axis as a promising therapeutic target for IS, offering precision-targeted immunomodulation strategies for IS.

## Supplementary Material

Supplementary figures.

## Abbreviations

IS: ischemic stroke
TIA1: T-cell intracellular antigen 1
SGs: stress granules
OGD: oxygen-glucose deprivation
IGF2: Insulin-like Growth Factor 2
CNS: central nervous system
ER: endoplasmic reticulum
RBPs: RNA binding proteins
CKO: conditional knockout
WT: wild-type
SPF: specific pathogen-free
HMC3: Human Microglia Clone 3
N2a: Neuro-2a
ATCC: American Type Culture Collection
PTS: Photothrombotic stroke model
mNSS: Modified neurological severity score
SDS-PAGE: sodium dodecyl sulphate polyacrylamide gel electrophoresis
CST: Cell Signaling Technology
HRP: horseradish peroxidase
TTC: 2, 3, 5-triphenyltetrazolium chloride
TUNEL: TdT-mediated dUTP Nick-End Labeling
LSCI: Laser speckle contrast imaging
CBF: cerebral blood flow
ROI: region of interest
Pus: perfusion units
RT-PCR: Real-Time PCR
qPCR: quantitative real-time PCR
*Ifn-γ*: interferon-γ
*Il-1β*: interleukin-1β
*Il-6*: interleukin-6
*Il-4*: interleukin-4
*Tgf-β*: transforming growth factor-β
DAMPs: damage-associated molecular patterns
hESCs: human embryonic stem cells
EAE: experimental autoimmune encephalomyelitis
DSS: dextran sodium sulfate
