# Supplementary material for: Microglial TIA1-mediated stress granules promote neuroinflammation and aggravate neuron loss in mice after ischemic stroke by inhibiting IGF2 signaling
Source: Theranostics. 2026 Jan 1;16(5):2627–48. doi: 10.7150/thno.122008 (PMC12713190; doi:10.7150/thno.122008)
Supplement: Supplementary file 1 — Supplementary figures. [file thnov16p2627s1.pdf]

Supplementary materials

Figure S1. Schematic of PTS.

(A) Schematic of neurobehavioral detection and photothrombosis (PTS). (**B**) Representative images of TTC staining of WT mice on 3rd day after IS.

Figure S2. Conditional deletion of microglial TIA1 did not affect the development or motor function of mice.

(**A**-**B**) Genotyping of *Tia1*$^{\text{Cx3cr1}}$-CKO mice. (**C**) Immunostaining analysis of TIA1 (red) and Iba1 (green) in the infarct area of *Tia1*$^{\text{f/f}}$ and *Tia1*$^{\text{Cx3cr1}}$-CKO mice on 3rd day after IS. Scale bar, 20 μm. (**D**) Representative images of 2-month-old *Tia1*$^{\text{f/f}}$ and *Tia1*$^{\text{Cx3cr1}}$-CKO mice. (**E**) Quantitative analysis of body weight of *Tia1*$^{\text{f/f}}$ mice and *Tia1*$^{\text{Cx3cr1}}$-CKO mice (n = 8 mice per group). (**F**) Representative tracing images of the open field test of *Tia1*$^{\text{f/f}}$ and *Tia1*$^{\text{Cx3cr1}}$-CKO mice. (**G**) Quantitative analysis of the total moved distance of *Tia1*$^{\text{f/f}}$ and *Tia1*$^{\text{Cx3cr1}}$-CKO mice in the open field test as shown in (**F**) (n = 7 mice per group). (**H**-**I**) Behavioral analysis of *Tia1*$^{\text{f/f}}$ and *Tia1*$^{\text{Cx3cr1}}$-CKO mice by cylinder test (**H**) (n = 7 mice in the *Tia1*$^{\text{f/f}}$ group and n = 6 mice in the *Tia1*$^{\text{Cx3cr1}}$-CKO group) and rotarod test (**I**) (n = 7 mice per group). Data were shown as mean ± s.e.m. *ns=no significance*, compared with *Tia1*$^{\text{f/f}}$ group.

Figure S3. TIA1 was upregulated in microglia after OGD, and both *Tia1*-overexpressing and *Tia1*-knockdown HMC3 cells were established.

(**A**) Western blotting analysis of TIA1 expression in HMC3 cells after OGD. (**B**) Western blotting analysis of TIA1 expression in control and *Tia1*-knockdown HMC3 cells. (**C**) Immunostaining analysis of TIA1 (red) and EGFP (green) in control and *Tia1*-knockdown HMC3 cells after OGD. (**D**) Western blotting analysis of TIA1 expression in control and *Tia1*-overexpressing HMC3 cells. (**E**) Immunostaining analysis of TIA1 (red) and EGFP (green) in control and *Tia1*-overexpressing HMC3 cells after OGD. (**F**) Quantitative analysis of TIA1 expression as shown in (**A**) (normalized to the mean expression of control group, n = 3 per group). (**G**) Quantitative analysis of the relative expression of TIA1 in EGFP^{+} cells as shown in (**C**) (n = 50 cells per group). (**H**)

Quantitative analysis of the relative expression of TIA1 in EGFP$^+$ cells as shown in (**E**) (n = 50 per group). Scale bars, 10 μm. Data were shown as mean ± s.e.m. $^{**}P < 0.01$, compared with the control group.

Figure S4. *TIA1*-overexpression promoted the inflammatory responses, and inhibited microglial phagocytic activity and increased neuronal death after OGD. (**A**) Western blotting analysis of CD206 and CD86 expression in control or *Tia1*-overexpressing HMC3 cells after OGD. (**B**-**C**) Quantitative analysis of the relative expression of CD206 (**B**) and CD86 (**C**) as shown in (**A**) (normalized to the mean expression of control group, n = 3 per group). (**D**-**G**) Quantitative analysis of the relative mRNA levels of *Il-1β* (**D**), *Il-6* (**E**), *Il-4* (**F**) and *Tgf-β* (**G**) in control and *Tia1*-overexpressing HMC3 cells after OGD (normalized to the mean expression of control group, n = 4 per group (*IL-6*) and n = 3 in the all other groups). (**H**) Representative image of phagocytosis analysis after OGD in control and *Tia1*-overexpressing HMC3 cells. Scale bar, 10 μm. (**I**) Immunostaining analysis of LAMP1 (red) and EGFP (green) in control and *Tia1*-overexpressing HMC3 cells after OGD. Scale bar, 10 μm. (**J**) Immunostaining analysis of NeuN (red) and cleaved caspase3 (C-C3, green) of N2a cells after cocultured with control and *Tia1*-overexpressing HMC3 cells after OGD. Scale bars, 100 μm. (**K**) Quantitative analysis of the number of fluorescent beads per cell as shown in (**H**) (n = 18 per group). (**L**) Quantitative analysis of the relative expression of LAMP1 in EGFP$^+$ cells as shown in (**I**) (normalized to the mean expression of control group, n = 50 per group). (**M**) Quantitative analysis of the percentages of C-C3$^+$ cells in total NeuN$^+$ cells as shown in (**J**) (n = 10 per group). Data were shown as mean ± s.e.m. $^{*}P < 0.05$, $^{**}P < 0.01$, compared with the control group.

Figure S5. Inhibition of IGF2 aggravated the neuroinflammatory responses in *Tia1*^{Cx3cr1}-CKO mice after IS.

(**A**) Immunostaining analysis of IGF2 (green) and Iba1 (red) in infarct area of *Tia1*^{Cx3cr1}-CKO mice on the 3rd day after chromeceptin treatment following IS injury. (**B**) Quantitative analysis of the relative expression of IGF2 as shown in (**A**) (normalized to

the mean expression of sham group, n = 15 per group). (**C-G**) Quantitative analysis of the relative mRNA levels of *Ifn-γ* (**C**), *Il-1β* (**D**), *Il-6* (**E**), *Il-4* (**F**), *Tgf-β* (**G**) of *Tia1*^{Cx3cr1}-CKO mice on the 3rd day after chromeceptin treatment following IS injury (normalized to the mean expression of control group, n = 5 in the vehicle group and the chromeceptin group (*Il-1β*), n = 6 in the all other groups). Scale bars, 20 μm. Data were shown as mean ± s.e.m. $^{*}P < 0.05$, $^{**}P < 0.01$, compared with the Vehicle or Sham group.

Figure S6. IGF2 modulated microglial polarization and promoted LAMP1 expression in primary cultured microglia after OGD.

(**A**) Western blotting analysis of CD206 and CD86 in primary cultured *Tia1*$^{+/+}$ microglia under control, OGD, or OGD + IGF2 (20 ng/mL) conditions. (**B-C**) Quantitative analysis of relative CD206 (**B**) and CD86 (**C**) protein levels as shown in (**A**) (n = 3 per group). (**D**) Immunostaining analysis of LAMP1 (red), Iba1 (green), and DAPI (blue) in primary *Tia1*$^{+/+}$ microglia under control, OGD, or OGD + IGF2 (20 ng/mL) conditions. (**E**) Quantitative analysis of relative LAMP1 expression in Iba1^{+} cells as shown in (**D**) (n = 50 cells per group). Scale bars, 20 μm. Data were presented as mean ± s.e.m. *ns = no significance,* $^{*}P < 0.05$, $^{**}P < 0.01$; compared with the control or OGD group.

Figure S1

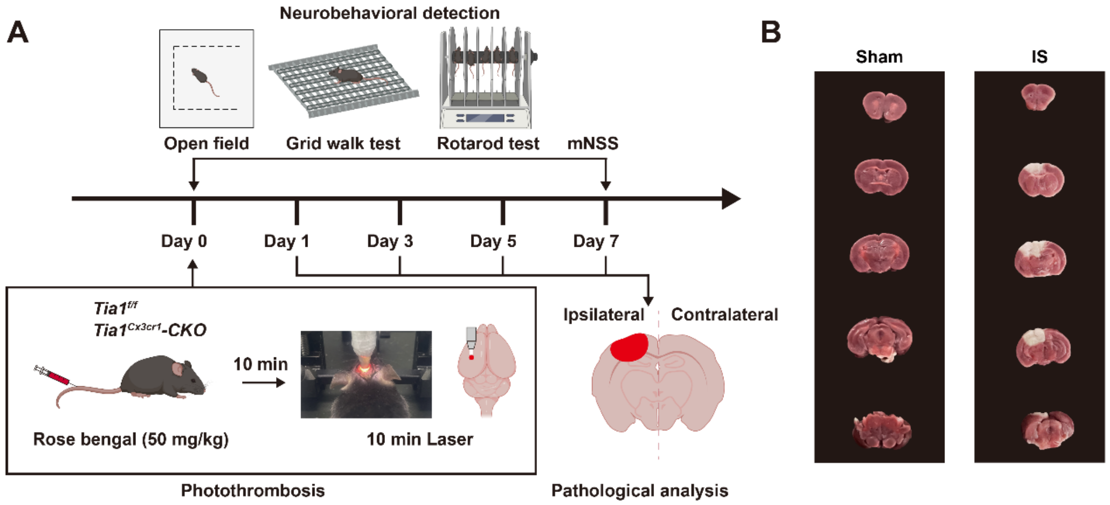
A
Neurobehavioral detection
Open field
Grid walk test
Rotarod test
mNSS
Day 0
Day 1
Day 3
Day 5
Day 7
Tia1$^{f/f}$
Tia1^{Cx3cr1}*-CKO*
10 min
Rose bengal (50 mg/kg)
10 min Laser
Photothrombosis
Ipsilateral
Contralateral
Pathological analysis
B
Sham
IS

Figure S2

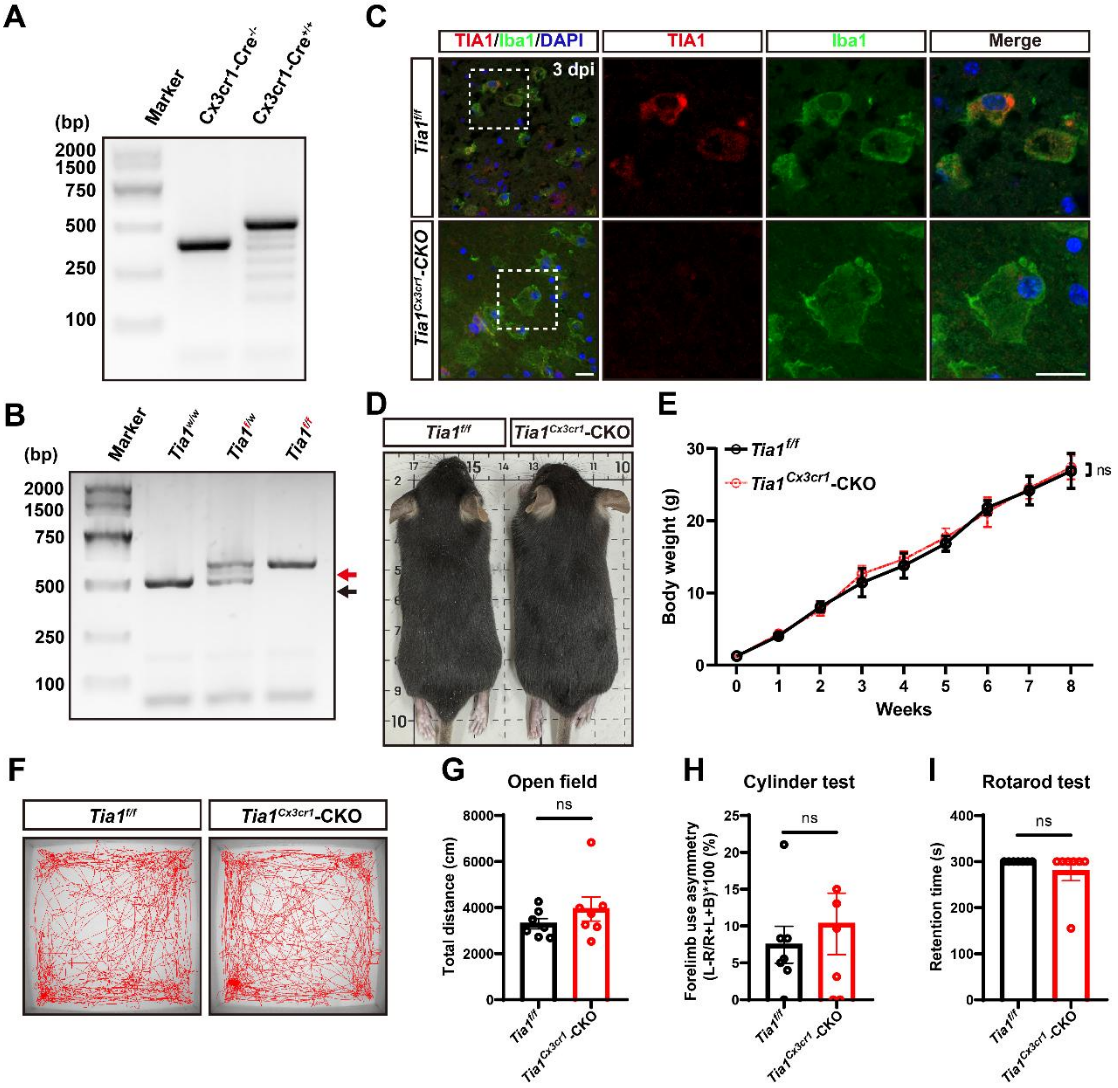

A
Marker
Cx3cr1-Cre-/-
Cx3cr1-Cre+/+
(bp)
2000
1500
750
500
250
100
B
Marker
Tia1w/w
Tia1f/w
Tia1f/f
(bp)
2000
1500
750
500
250
100
C
TIA1/Iba1/DAPI
TIA1
Iba1
Merge
3 dpi
Tia1f/f
Tia1Cx3cr1-CKO
D
Tia1f/f
Tia1Cx3cr1-CKO
E
Tia1f/f
Tia1Cx3cr1-CKO
ns
Body weight (g)
Weeks
F
Tia1f/f
Tia1Cx3cr1-CKO
G
Open field
Total distance (cm)
ns
H
Cylinder test
Forelimb use asymmetry (L-R/R+L+B)*100 (%)
ns
I
Rotarod test
Retention time (s)
ns

Figure S3

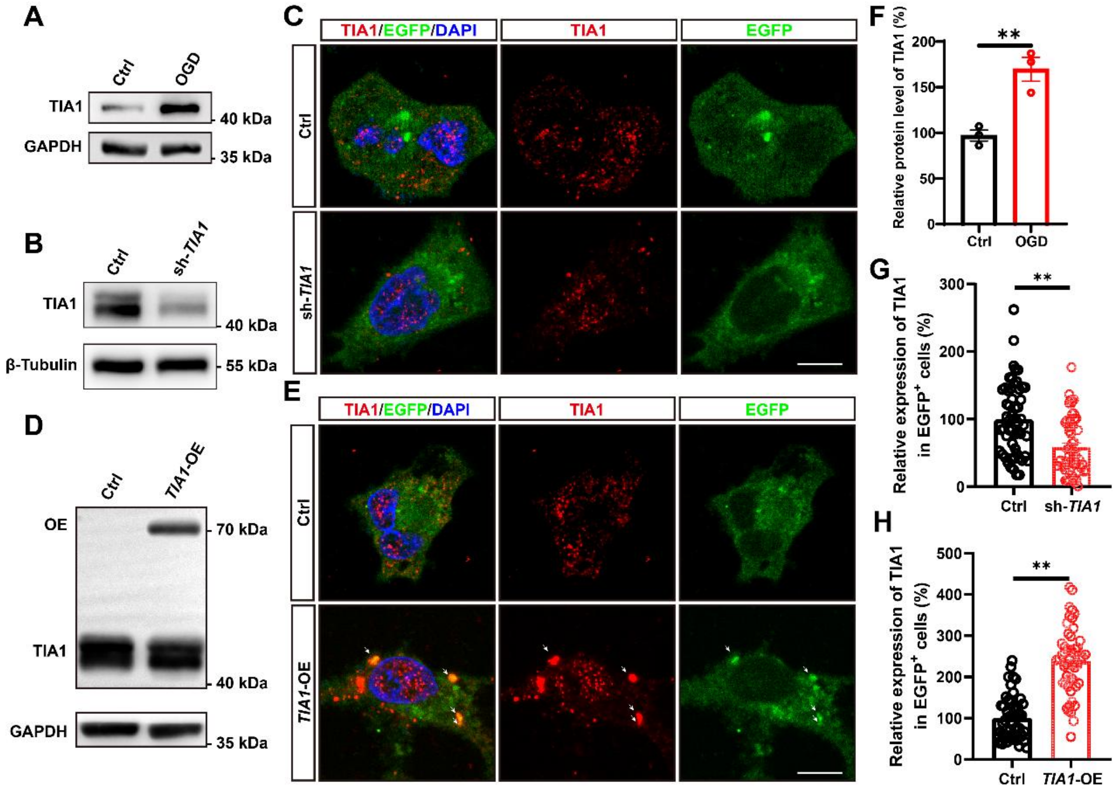
A
Ctrl
OGD
TIA1
GAPDH
40 kDa
35 kDa
B
Ctrl
sh-TIA1
TIA1
β-Tubulin
40 kDa
55 kDa
D
Ctrl
TIA1-OE
OE
TIA1
GAPDH
70 kDa
40 kDa
35 kDa
C
TIA1/EGFP/DAPI
TIA1
EGFP
Ctrl
sh-TIA1
E
TIA1/EGFP/DAPI
TIA1
EGFP
Ctrl
TIA1-OE
F
Relative protein level of TIA1 (%)
**
Ctrl
OGD
G
Relative expression of TIA1 in EGFP+ cells (%)
**
Ctrl
sh-TIA1
H
Relative expression of TIA1 in EGFP+ cells (%)
**
Ctrl
TIA1-OE

Figure S4

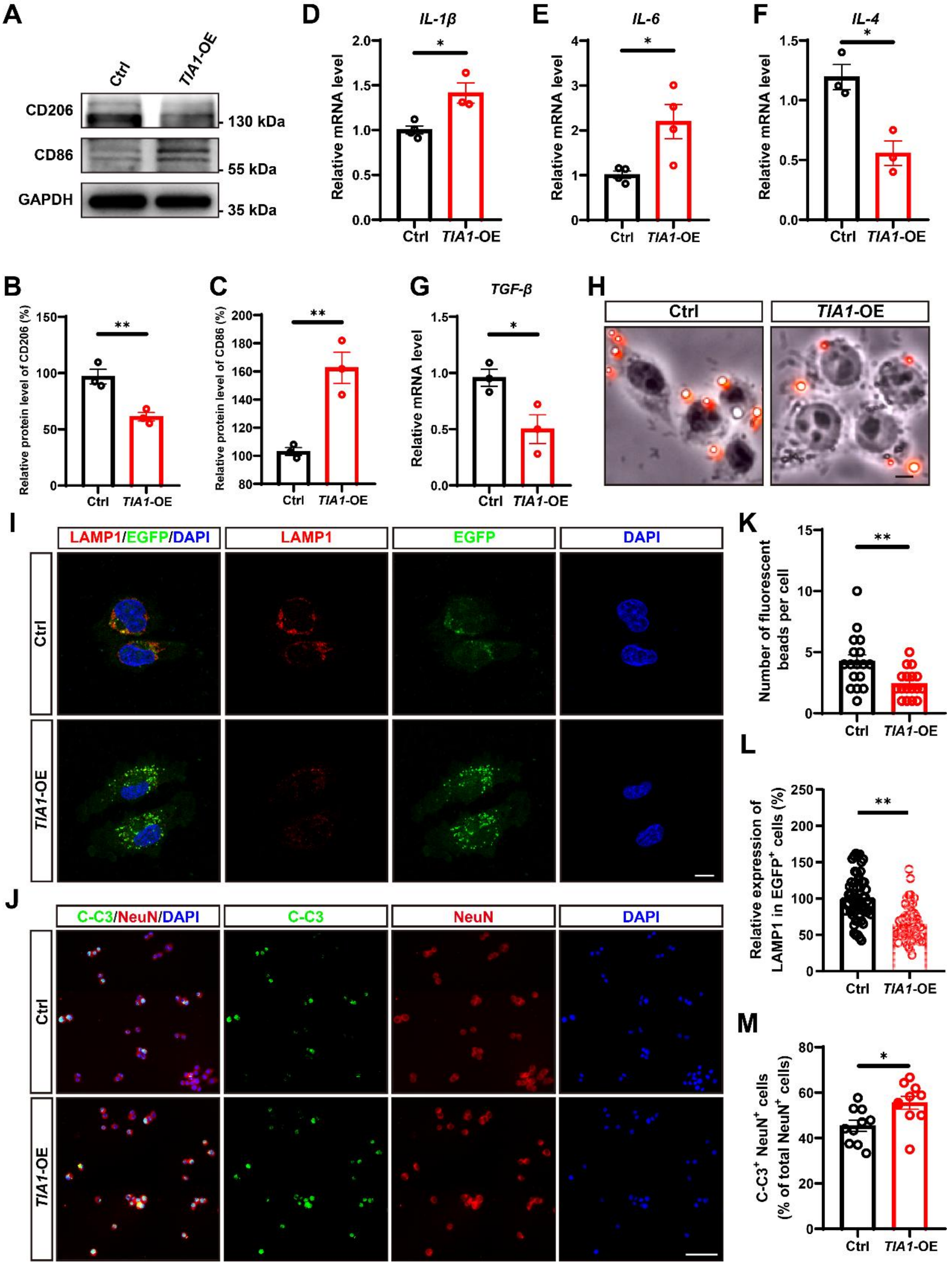

A
Ctrl
TIA1-OE
CD206
130 kDa
CD86
55 kDa
GAPDH
35 kDa
B
Relative protein level of CD206 (%)
C
Relative protein level of CD86 (%)
D
IL-1β
E
IL-6
F
IL-4
G
TGF-β
Relative mRNA level
H
I
LAMP1/EGFP/DAPI
LAMP1
EGFP
DAPI
K
Number of fluorescent beads per cell
L
Relative expression of LAMP1 in EGFP+ cells (%)
J
C-C3/NeuN/DAPI
C-C3
NeuN
M
C-C3+ NeuN+ cells (% of total NeuN+ cells)

Figure S5

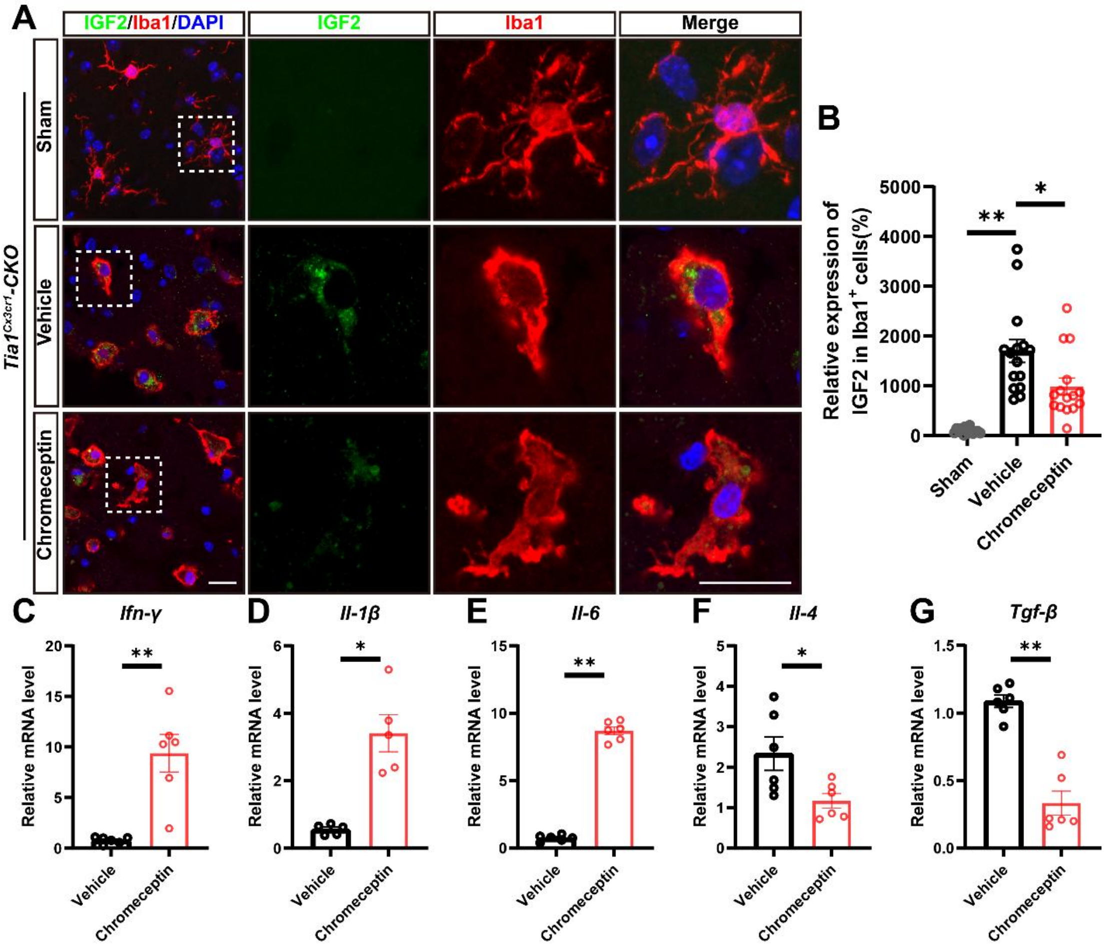

A
IGF2/Iba1/DAPI
IGF2
Iba1
Merge
Sham
Vehicle
Chromeceptin
Tia1Cx3cr1-CKO
B
Relative expression of IGF2 in Iba1+ cells(%)
5000
4000
3000
2000
1000
0
**
*
Sham
Vehicle
Chromeceptin
C
Ifn-γ
D
Il-1β
E
Il-6
F
Il-4
G
Tgf-β
Relative mRNA level
Vehicle
Chromeceptin

97 **Figure S6**

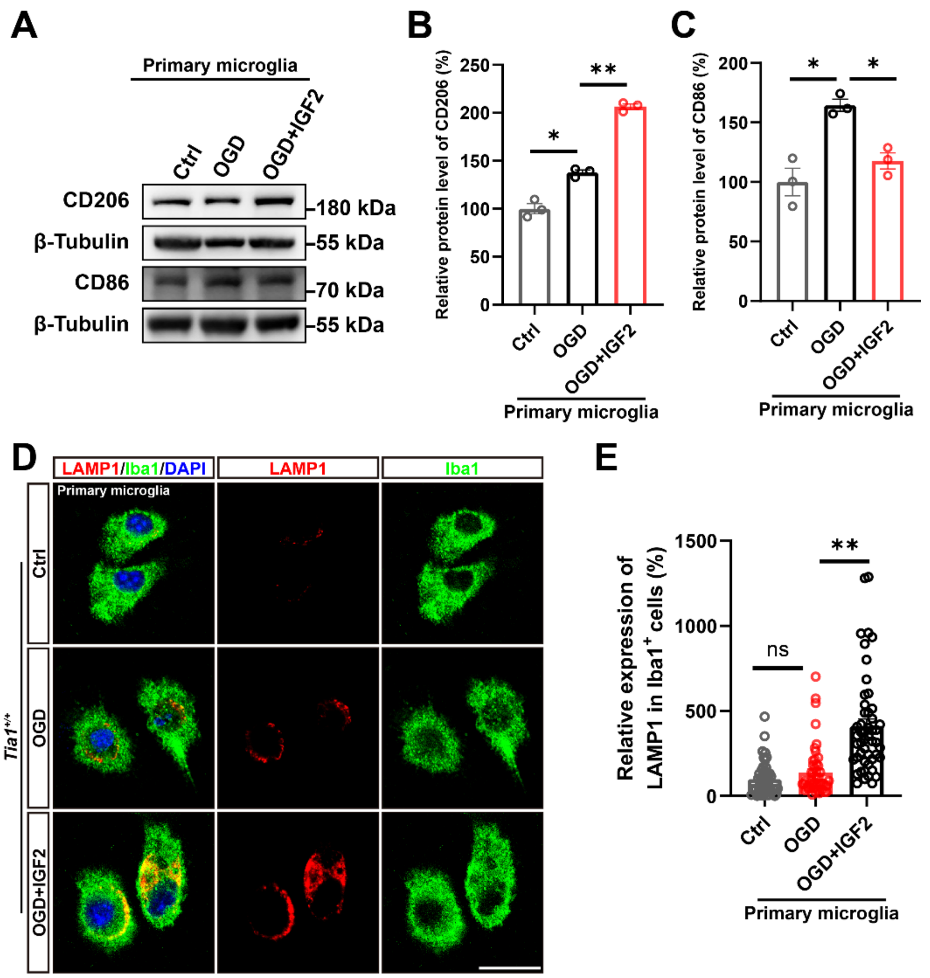

98
